# Targeted temperature management alleviates post-resuscitation myocardial dysfunction by inhibiting ferroptosis

**DOI:** 10.1038/s41420-025-02356-5

**Published:** 2025-02-21

**Authors:** Yingying Zhang, Weiwei Du, Ting Kong, Tianfeng Hua, Honghao Ma, Yan Hu, Sinong Pan, Bingrui Ling, Min Yang, Cheng Cheng

**Affiliations:** 1 Department of Cardiology, the Second Affiliated Hospital of Anhui Medical University, Hefei, 230601 Anhui China; 2 The Laboratory of Cardiopulmonary Resuscitation and Critical Care Medicine, the Second Affiliated Hospital of Anhui Medical University, Hefei, 230601 Anhui China; 3 Department of General Practice Department, the Second Affiliated Hospital of Anhui Medical University, Hefei, 230601 Anhui China; 4 The Second Department of Intensive Care Unit, The Second Affiliated Hospital of Anhui Medical University, Hefei, 230601 Anhui China; 5 Department of Cardiology, the First Affiliated Hospital of Anhui Medical University, Hefei, 230088 Anhui China

**Keywords:** Experimental models of disease, Cardiovascular diseases

## Abstract

Targeted temperature management (TTM) is a vital intervention for cardiac arrest survivors to mitigate post-resuscitation myocardial dysfunction (PRMD). However, the optimal temperature for TTM remains a topic of debate. This study investigates the effects of TTM at different temperatures and explores the underlying mechanisms using in vivo and in vitro models of myocardial ischemia/reperfusion (I/R) injury following cardiac arrest (CA) and cardiopulmonary resuscitation (CPR). We found that TTM at 33 °C significantly improved post-resuscitation hemodynamics and myocardial function, reducing both myocardial and mitochondrial damage in the rat model of CA/CPR. Additionally, Deferoxamin (DFO), as an iron chelating agent, also demonstrated protective effects against PRMD. Both in vitro and in vivo experiments confirmed that hypothermia at 33 °C and DFO mitigated mitochondrial damage, oxidative stress, lipid peroxidation, and iron overload, while suppressing ferritinophagy and ferroptosis. Furthermore, TTM at 33 °C and DFO facilitated the nuclear translocation of nuclear factor erythroid 2-related factor 2 (Nrf2), with Nrf2 activation leading to inhibited ferritinophagy and enhanced iron export. Our findings indicate that TTM at 33° C, as opposed to 36° C, significantly alleviates PRMD and reduced myocardial damage by inhibiting ferroptosis. Theses protective effects are associated with Nrf2 activation and modulation of iron homeostasis. Moreover, DFO not only suppressed ferroptosis through its iron chelation properties but also by activating the Nrf2 axis.

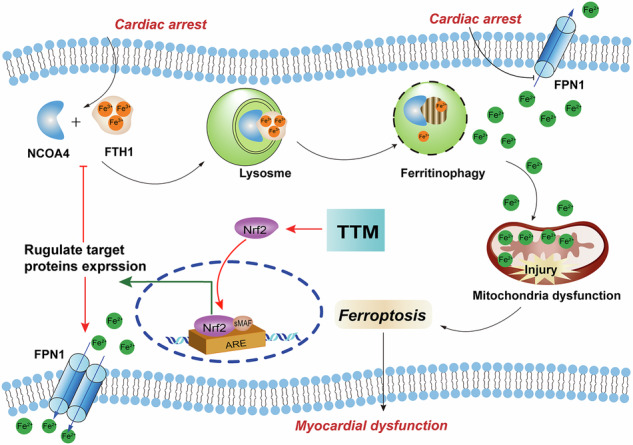

## Introduction

Cardiac arrest (CA) is a significant public health concern associated with high rates of disability and mortality. Despite advances in resuscitation and critical care, the survival rate among CA patients who return to spontaneous circulation (ROSC) remains unacceptably low [1, 2]. Post-resuscitation myocardial dysfunction (PRMD) is a leading cause of early death following resuscitation. Its pathophysiology, which likely involves global myocardial ischemia/reperfusion (I/R) injury, mitochondrial damage, and activation of antioxidant stress pathways, remains complex and poorly understood [3].

Current guidelines recommend maintaining post-arrest temperature control between 32 °C – 37.5 °C to optimize outcomes [4]. Targeted temperature management (TTM) in mild hypothermia has shown promise in improving cardiac function based on clinical data and animal studies [5–7]. However, the precise protective mechanisms of TTM against PRMD remain unclear, and optimal target temperature remains a subject of debate.

Ferroptosis is a recently recognized form of programmed cell death distinct from apoptosis, necrosis, and autophagy. It is characterized by iron overload, excessive accumulation of lipid peroxides, mitochondrial membrane rupture and altered mitochondrial morphology [8–10]. Emerging evidence suggests ferroptosis plays a pivotal role in myocardial I/R injury and PRMD [11–13]. Intracellular iron homeostasis, governed by processes such as ferritin synthesis/degradation and iron export via ferroportin, is critical in regulating ferroptosis. Nuclear receptor coactivator 4 (NCOA4), a crucial cargo receptor, specifically mediates ferritinophagy, influencing iron levels and potentially exacerbating iron overload [8, 14, 15].

Given ferroptosis’s involvement in myocardial injury post-CA, there is growing interest in targeting TTM as a therapeutic strategy for PRMD. Nuclear factor erythroid 2-related factor 2 (Nrf2), a transcription factor, plays a pivotal role in regulating genes involved in iron metabolism and ferroptosis.[16–18] Studies have indicated that hypothermic therapy post-ROSC can attenuate neuronal damage in CA models via the Nrf2 pathway [19, 20]. We hypothesize that TTM mitigates PRMD through mechanisms involving Nrf2 and its downstream effectors.

In this study, we aimed to investigate how TTM affects PRMD and iron homeostasis in the context of ferroptosis using a rat model of CA/CPR. Additionally, we explored how different temperatures of hypothermia during reoxygenation influence ferroptosis in cardiomyocytes subjected to hypoxia-reoxygenation (H/R).

## Results

### TTM-33° C improved post-resuscitation hemodynamics and myocardial function in rats

**Fig. 1:**
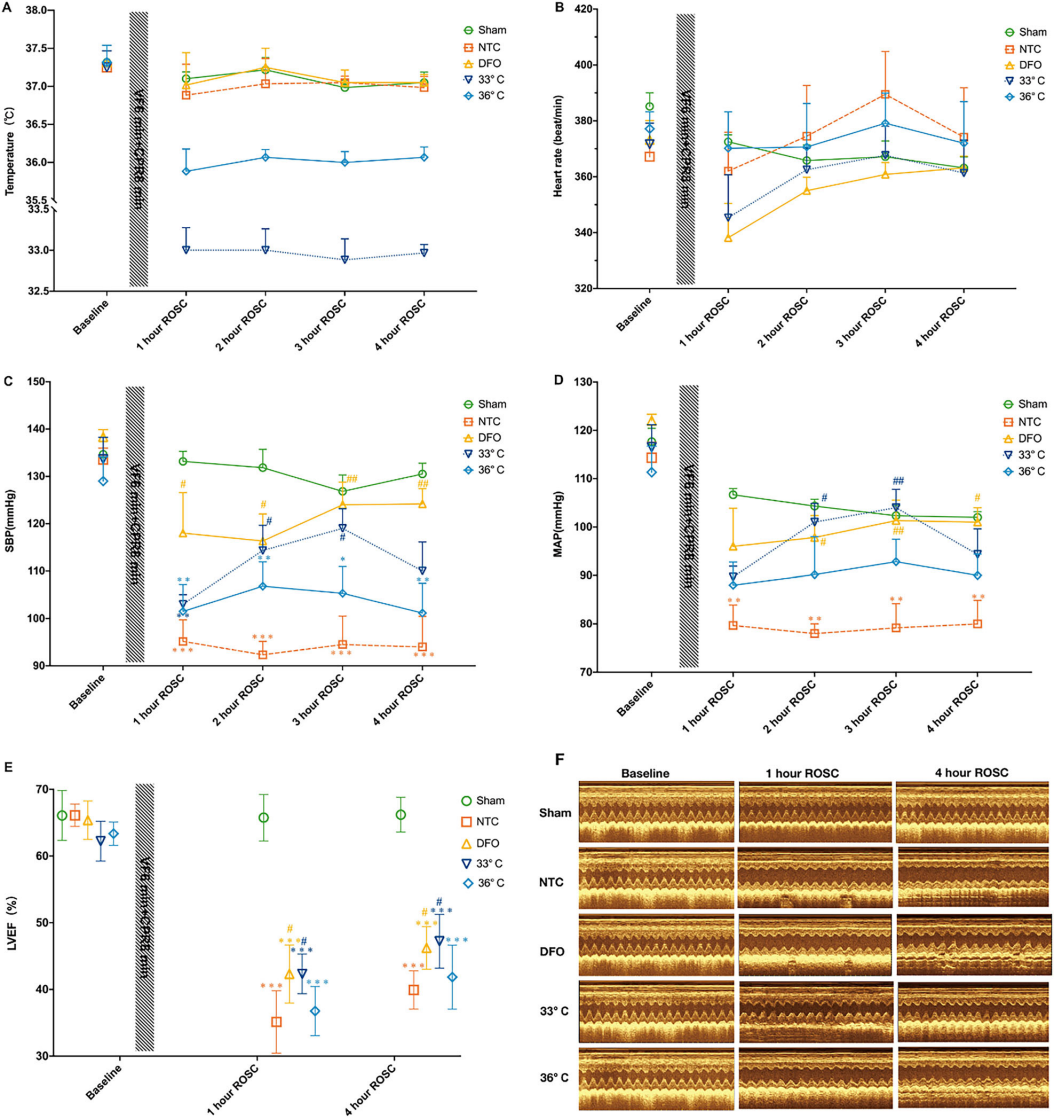
Changes in physiological parameters at various time points post-resuscitation. **A** Temperature; **B** heart rate; **C** Systolic Blood Pressure (SBP); **D** Mean Arterial Pressure (MAP); **E** Left Ventricular Ejection Fraction (LVEF); **F** Left Ventricular Function; Changes in body temperature, heart rate, SBP, MAP, LVEF at baseline, 1 h, 2 h, 3 h, and 4 h post-resuscitation in each group. Sham represents the sham-operated group, NTC represents the normothermic control group, DFO represents the deferoxamine group, and 33°C and 36 °C represent the different temperature groups. Data are presented as mean ± standard deviation. (*n* = 6 in each group). *Indicates a significant difference compared to the Sham group, # indicates a significant difference compared to the NTC group.

Baseline characteristics of body weight, temperature, heart rate, SBP and MAP did not significantly differ among the five experimental groups. All animals subjected to epicardial fibrillation were successfully resuscitated and survived the 4 h post-resuscitation monitoring period. The core temperature was maintained at a certain level for 4 h after ROSC (Fig. 1A). There were no significant differences in heart rate among the groups during the ROSC period (Fig. 1B). However, animals treated with TTM at 33 °C and DFO exhibited higher SBP and MAP levels compared to those in the NTC group at 2 h and 3 h ROSC (Fig. 1C, D). Left ventricular EF impairment was notably improved with DFO and TTM at 33 °C treatments compared to the NTC group (Fig. 1E, F). There were no significant differences in EF between the TTM at 36 °C and NTC groups at both 1 h or 4 h ROSC time points (Fig. 1E).

### TTM-33° C alleviates myocardial damage and mitochondrial injury in the rat model of CA/CPR

**Fig. 2:**
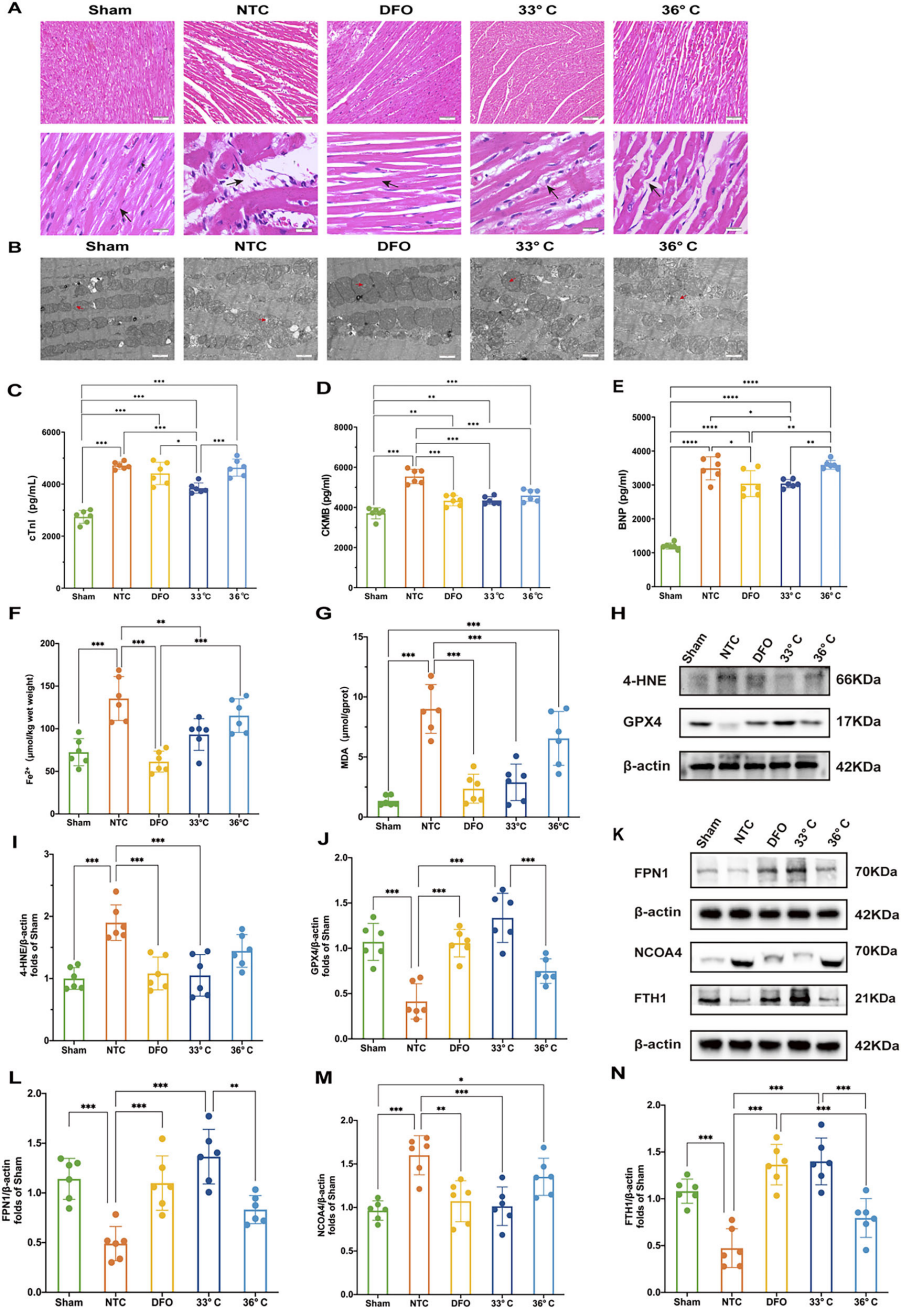
Histological and biochemical analysis of myocardial tissues post-resuscitation in different treatment groups. **A** Hematoxylin and Eosin (H&E) staining of myocardial tissue. Scale bar:50 μm. **B** Transmission electron microscopy images of myocardial ultrastructure. Scale bar:5 μm. **C**–**E** Levels of cardiac injury biomarkers: CTNI, CK-MB, and BNP. **F**, **G** Oxidative stress markers: Iron and MDA levels. **H** Western blot of oxidative stress-related proteins: 4-HNE and GPX4 protein levels are assessed; **I**–**N** Protein expression analysis: The expressions of 4-HNE, GPX4, FPN1, NCOA4, and FTH1 in myocardial tissue. (*n* = 6) **K** Western blot validation of FPN1, NCOA4, and FTH1 proteins, supporting the quantitative analysis. β-actin serves as a loading control. Data are presented as mean ± SD. (*n* = 6 in each group). *p < 0.05, **p < 0.01, ***p < 0.001.

At 4 h ROSC, histological examination of HE-stained left ventricular sections from all groups revealed cardiomyocyte disarrangement and swelling, partial myocytolysis, and a loosened extracellular matrix compared to the sham group. Treatment with either TTM at 33 °C or 36 °C, as well as DFO, effectively mitigated swelling and myocytolysis to varying degrees following CPR (Fig. 2A). Transmission electron microscopy of the control group showed mitochondrial shrinkage, outer membrane rupture, and loss or reduction of lamellar cristae. Conversely, treatment with TTM or DFO resulted in less damage to mitochondrial membranes and preserved lamellar cristae structure (Fig. 2B). Levels of cTnI, CK-MB, and BNP in myocardial tissue were significantly elevated 4 h post-resuscitation in the CA/CPR model groups compared to the sham group. However, treatment with TTM at 33 °C and DFO markedly reduced cTnI, CK-MB, and BNP levels compared to the NTC group (Fig. 2C–E).

### TTM-33° C reduced the levels of ferrous iron and inhibited ferroptosis via ferritinophagy and iron export in the rat model of CA/CPR

Iron overload and lipid peroxidation are critical hallmarks of ferroptosis. To assess the impact of TTM on ferroptosis, we measured levels of Fe^2+^, MDA, and 4-hydroxynonenal (4-HNE) in myocardial tissues. Compared to the NTC group, the TTM at 33 °C group showed a significant reduction in Fe^2+^ levels (Fig. 2F). Additionally, treatment with DFO markedly decreased ferrous iron levels (Fig. 2F).

Following CA and subsequent resuscitation, myocardial MDA levels significantly increased compared to sham-operated animals. Both TTM at 33 °C and DFO treatment effectively reduced MDA levels in resuscitated rats (Fig. 2G). The expression of 4-HNE proteins, indicative of lipid peroxidation, was also substantially elevated post-CA (Fig. 2H, I). However, treatment with TTM at 33 °C and DFO significantly decreased the content of 4-HNE-modified proteins in resuscitated rats (Fig. 2H, I).

Glutathione peroxidase 4 (GPX4) is a key regulator of ferroptosis. Resuscitation following CA led to a significant reduction in GPX4 expression in the NTC group compared to sham-operated animals. Remarkably, TTM at 33 °C and DFO treatment markedly increased GPX4 expression (Fig. 2H, J). These findings suggest that TTM at 33 °C can attenuate myocardial ferroptosis by mitigating iron overload and lipid peroxidation in our rat model of CA/CPR.

Ferroportin 1 (FPN1) is the primary iron-export protein in mammals, essential for maintaining systemic iron balance. We observed a significant reduction in FPN1 expression following resuscitation, which was effectively reversed by treatment with DFO or TTM at 33 °C (Fig. 2K, L). Ferritinophagy plays a critical role in modulating intracellular labile iron pools by facilitating the degradation of ferritin, the major iron storage protein complex consisting of ferritin light polypeptide 1 (FTL1) and ferritin heavy polypeptide 1 (FTH1). Nuclear receptor coactivator 4 (NCOA4) is a crucial cargo receptor that specifically targets ferritin to autophagosomes for lysosomal degradation and subsequent iron release [14]. We assessed NCOA4 and FTH1 levels in a rat model of CA/CPR. Post-resuscitation, NCOA4 expression was markedly elevated compared to sham-operated animals. Treatment with DFO and TTM at 33 °C significantly reduced NCOA4 levels (Fig. 2K, M). Moreover, TTM at 33 °C and DFO treatment attenuated the resuscitation-induced degradation of FTH1 (Fig. 2K, N). However, the mechanisms through which TTM regulates the NCOA4 and FTH1 interaction remain to be elucidated.

### Hypothermia at 33 °C alleviated H/R injury in vitro

**Fig. 3:**
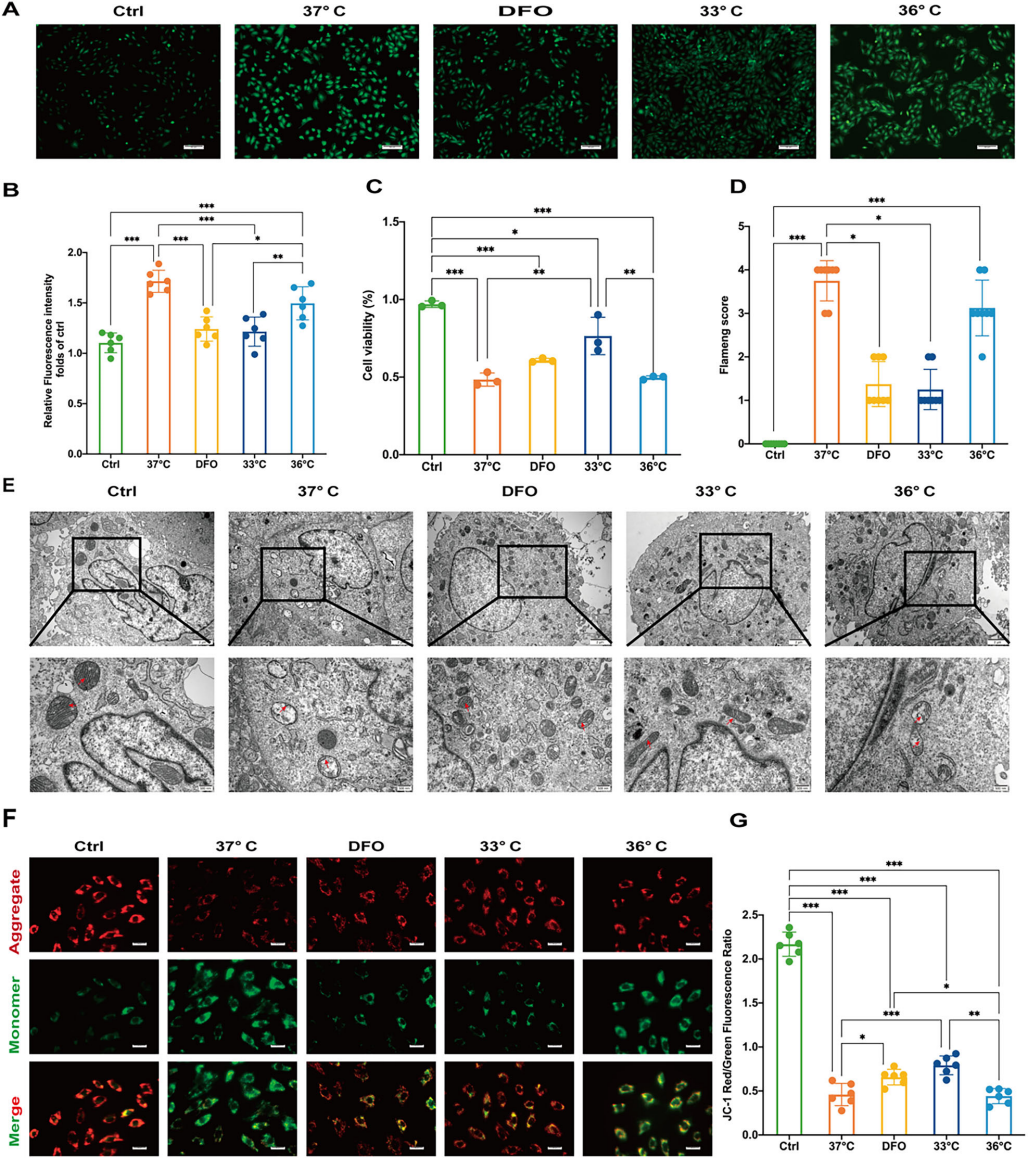
Hypothermia at 33 °C alleviated H/R injury in vitro. **A** Fluorescent microscopy of ROS. Scale bar:50 μm. **B** Relative fluorescence intensity of ros. **C** Cell viability assay. **D** Flameng score. **E** Transmission electron microscopy images of cell ultrastructure. Scale bar:500 nm. **F** Fluorescent microscopy of JC-1 stained cells. Scale bar:100 μm. **G** JC-1 red/green fluorescence ratio. Data are presented as mean ± SD. (*n* = 6 in each group). *p < 0.05, **p < 0.01, ***p < 0.001.

To determine the optimal target temperature for TTM, we established a cardiomyocyte H/R model with different reoxygenation temperatures. Following H/R, cellular ROS levels markedly increased. Both hypothermia at 33 °C and treatment with DFO reduced ROS production (Fig. 3A, B). Cell viability was assessed using the CCK-8 assay. Hypothermia at 33 °C significantly mitigated impairment following H/R (Fig. 3C). TEM revealed varying degrees of outer mitochondrial membrane rupture and loss of lamellar cristae after H/R injury in H9c2 cells. Hypothermia at 33 °C and DFO treatments rescued these H/R-induced mitochondrial effects (Fig. 3D, E). Mitochondrial membrane potential, assessed by JC-1 staining, showed significant preservation with hypothermia at 33 °C following H/R-induced membrane potential loss. In contrast, this effect was not observed with DFO treatment or at 36°C (Fig. 3F, G).

### Hypothermia at 33 °C inhibited ferroptosis through disrupt NCOA4-FTH1 interaction in vitro

**Fig. 4:**
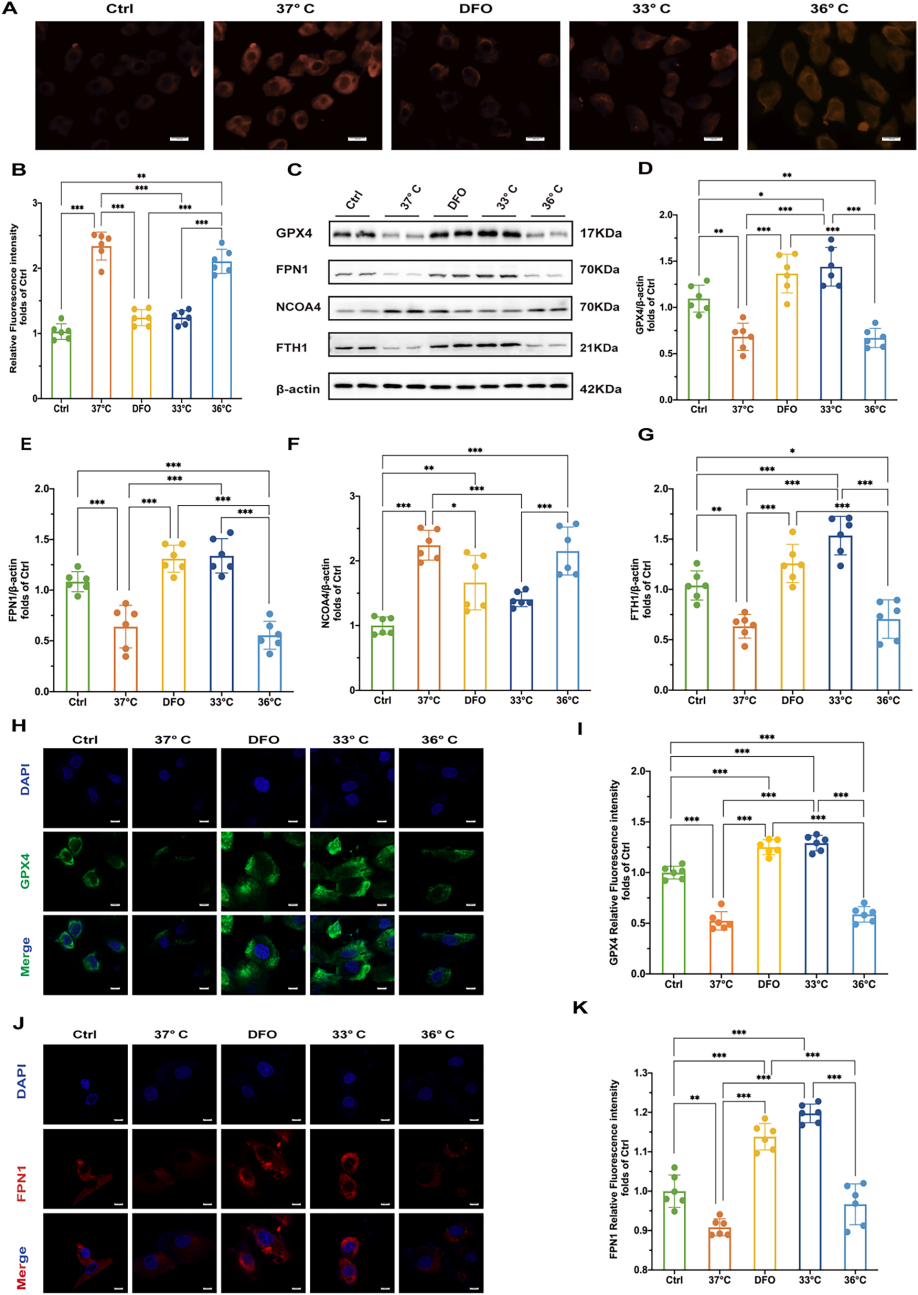
Quantitative and visual analysis of Fe^2+^ levels and key proteins in treated cells. **A** Fluorescence microscopy images of Fe^2+^ in cells. Scale bar:100μ m. **B** Relative fluorescence intensity of Fe^2+^. **C** Results of Western blot of GPX4, FPN1, NCOA4 and FTH1 proteins in cells of each treatment group. **D**–**G** Quantitative analysis of relative expression of GPX4, FPN1, NCOA4 and FTH1 proteins in cells of each treatment group (*n* = 6). **H** Immunofluorescence staining results of GPX4 in cells of each treatment group (DAPI staining showed cell nuclei). Scale bar:20 μm. **I** Quantitative analysis of GPX4 immunofluorescence intensity of cells in each treatment group. **J** FPN1 immunofluorescence staining results of cells in each treatment group (DAPI staining showed cell nuclei). **K** Quantitative analysis of FPN1 immunofluorescence intensity of cells in each treatment group. Statistical significance is expressed as: *p < 0.05, **p < 0.01, ***p < 0.001.

To explore the mechanisms underlying the protective effects of hypothermia during H/R injury, we measured levels of ferrous iron, proteins associated with ferroptosis, and ferritinophagy in H9c2 cardiomyocytes. Following H/R injury, fluorescence intensity of Fe^2+^ significantly increased. Hypothermia at 33 °C and treatment with DFO effectively reduced Fe^2+^ production, with superior protective effects compared to hypothermia at 36°C (Fig. 4A, B).

**Fig. 5:**
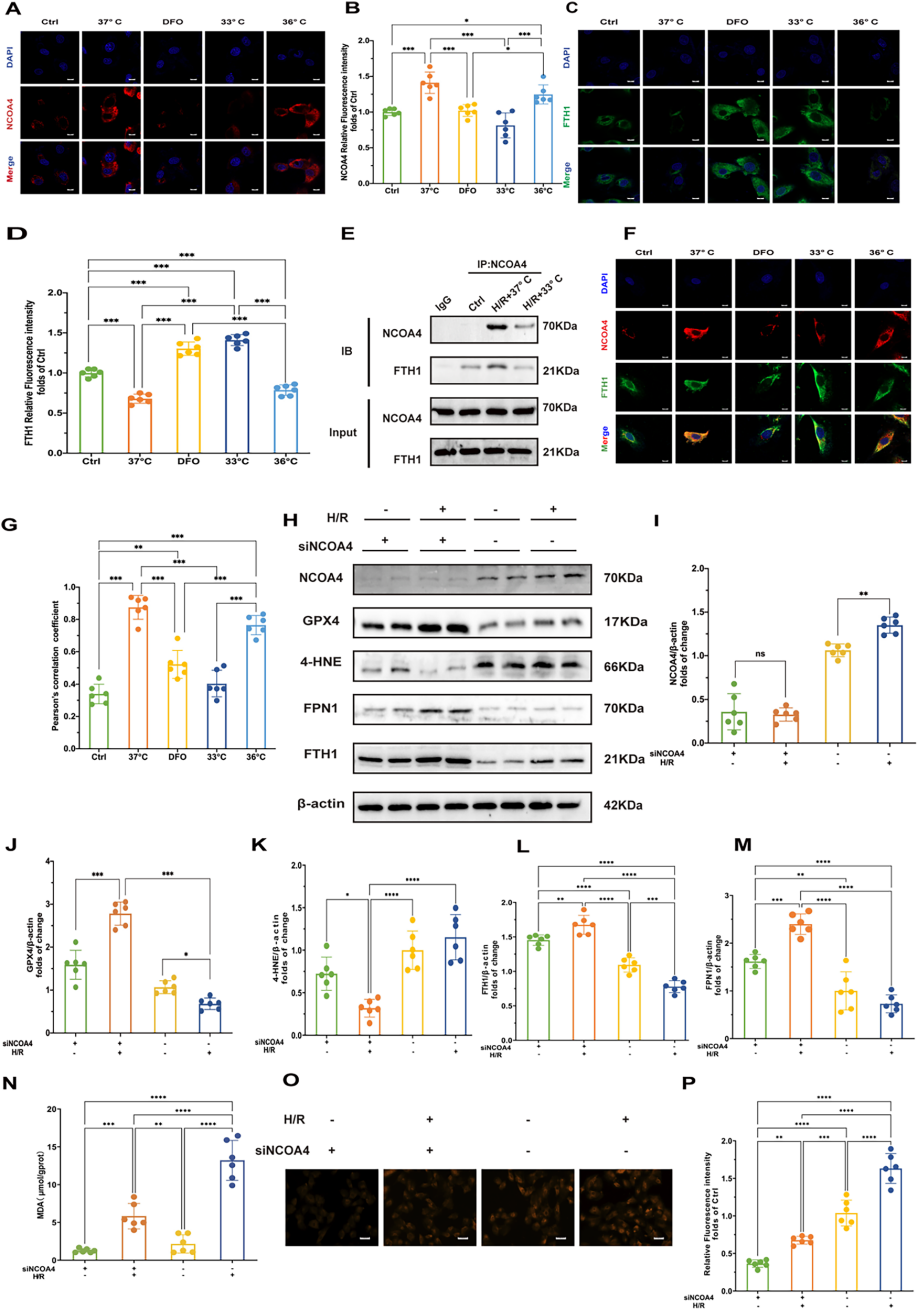
Hypothermia at 33 °C inhibited ferroptosis through disrupt NCOA4-FTH1 interaction. **A** Results of NCOA4 immunofluorescence staining of cells in each treatment group (DAPI staining shows cell nuclei). Scale bar:20 μm. **B** Quantitative analysis of NCOA4 immunofluorescence intensity of cells in each treatment group (*n* = 6). **C** Immunofluorescence staining results of FTH1 in cells of each treatment group (DAPI staining showed cell nuclei). Scale bar:20 μm. **D** Quantitative analysis of FTH1 immunofluorescence intensity of cells in each treatment group (*n* = 6). **E** Immunoprecipitation results of NCOA4 and FTH1 in cells of each treatment group. **F** Double immunofluorescence staining results of NCOA4 and FTH1 in cells of each treatment group. Scale bar:20 μm. **G** Quantitative analysis of co-localization coefficients of NCOA4 and FTH1 in cells of each treatment group (*n* = 6). **H** Immunoblot of NCOA4, GPX4, 4-HNE, FPN1, and FTH1 under H/R treatment with or without NCOA4 knockdown (siNCOA4). β-actin is the loading control. **I** Quantitative analysis of NCOA4 protein expression after H/R treatment and siNCOA4 interference (*n* = 6). **J** Quantitative analysis of GPX4 protein expression after H/R treatment and siNCOA4 interference (*n* = 6). **K** Quantitative analysis of 4-HNE protein expression after H/R treatment and siNCOA4 interference (*n* = 6). **L** Quantitative analysis of FTH1 protein expression after H/R treatment and siNCOA4 interference (*n* = 6). **M** Quantitative analysis of FPN1 protein expression after H/R treatment and siNCOA4 interference (*n* = 6). **N** Oxidative stress markers: MDA levels, reflecting oxidative stress levels under H/R and siNCOAeatment conditions. **O** Fluorescence microscopy images of Fe2+ under H/R treatment with or without NCOA4 knockdown (siNCOA4). Scale bar:100 μm. **P** Relative fluorescence intensity of Fe^2+^. Measurement data were presented as mean ± SD. Statistical significance is indicated as: *p < 0.05, **p < 0.01, ***p < 0.001.

Consistent with our animal experiments, hypothermia at 33 °C and DFO treatments increased levels of GPX4 and FPN1 after H/R injury compared to the 37°C group in vitro (Fig. 4C–E). In contrast, hypothermia at 36 °C did not significantly impact GPX4 or FPN1 expression (Fig. 4C–E). We also found that both 33 °C hypothermia and DFO treatment reduced NCOA4 levels (Fig. 4C, F), accompanied by an increase in FTH1 expression (Fig. 4C, G). Immunofluorescence assays further confirmed the effects of hypothermia at 33 °C and DFO on regulating GPX4, FPN1 and NCOA4 and FTH1 expression (Fig. 4H–K, Fig. 5A–D). These findings indicate that H/R injury induces ferrous iron overload and promotes ferroptosis. Hypothermia at 33 °C and DFO enhances iron export through increased FPN1 expression and decreases NCOA4 expression, thereby effectively regulating iron homeostasis and mitigating myocardial damage.

To further explore the interaction between NCOA4 and FTH1, we conducted co-IP assays and assessed immunofluorescence co-localization using Pearson’s correlation coefficient (PCC). Following H/R injury, there was a significant increase in the NCOA4-FTH1 interaction, as demonstrated by co-IP analysis (Fig. 5E). Immunofluorescence staining revealed a PCC R value significantly increased after H/R at 37°C, indicating enhanced co-localization. Both hypothermia at 33°C and DFO markedly reduced the PCC R value (Fig. 5F, G), suggesting that hypothermia and DFO disrupts the NCOA4-FTH1 interaction and inhibits ferritinophagy. These findings highlight that H/R injury enhances the interaction between NCOA4 and FTH1, promoting ferritinophagy. Conversely, hypothermia at 33°C and DFO attenuates this interaction, thereby potentially mitigating myocardial damage by inhibiting ferritinophagy.

To further establish the significance of NCOA4 in ferroptosis, we employed NCOA4 knockdown cells subjected to H/R injury. The results indicated that the knockdown of NCOA4 led to a notable increase in the levels of GPX4 and FTH1, while simultaneously decreasing the expression of 4-HNE (Fig. 5H–M). Additionally, the knockdown of NCOA4 resulted in a significant reduction in Fe²^+^ fluorescence intensity and a marked decrease in MDA levels. (Fig. 5N–P). This finding underscores the critical role of ferritinophagy in mediating H/R-induced cardiac ferroptosis.

### Hypothermia at 33°C promoted Nrf2 nuclear translocation to inhibited ferroptosis

**Fig. 6:**
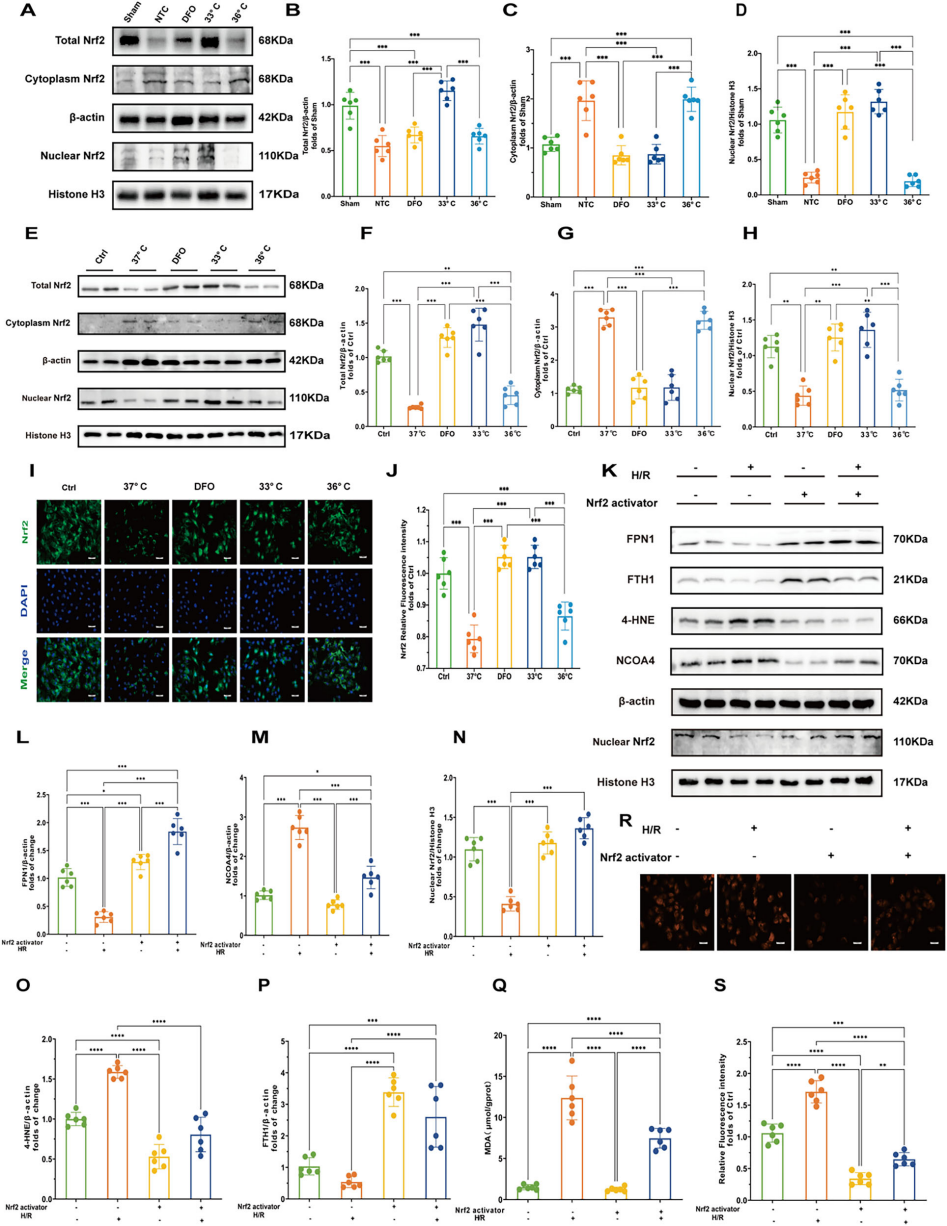
Hypothermia at 33 °C promoted Nrf2 nuclear translocation to inhibited ferroptosis. **A** Western blot of total Nrf2, cytoplasmic Nrf2, and nuclear Nrf2 proteins in myocardial tissues. **B**–**D** Quantification of total Nrf2, cytoplasmic Nrf2, and nuclear Nrf2 protein levels in myocardial tissues of each treatment group. (*n* = 6) **E** Western blot of total Nrf2, cytoplasmic Nrf2, and nuclear Nrf2 proteins in myocardial tissues in cells. **F**–**H** Quantification of total Nrf2, cytoplasmic Nrf2, and nuclear Nrf2 protein levels in cells. (*n* = 6) **I** Immunofluorescence staining of Nrf2 in different treatment groups (DAPI staining shows nuclei). Scale bar:20 μm. **J** Quantification of Nrf2 immunofluorescence intensity in different treatment groups. (*n* = 6) **K** Immunoblot analysis of FPN1, FTH1, 4-HNE,Nuclear Nrf2 and NCOA4 under hypoxia/reoxygenation (H/R) conditions with or without Nrf2 activation. β-actin and Histone H3 are used as loading controls. **L**–**P** Quantification of FPN1, 4-HNE,NCOA4,FTH1 and nuclear Nrf2 protein levels after H/R treatment and Nrf2 activator treatment. (*n* = 6) **Q** Oxidative stress markers:MDA levels, reflecting oxidative stress levels under hypoxia/reoxygenation (H/R) conditions with or without Nrf2 activation.**R** Fluorescence microscopy images of Fe2+ under hypoxia/reoxygenation (H/R) conditions with or without Nrf2 activation. **S** Relative fluorescence intensity of Fe^2+^. Measurement data were presented as mean ± SD. Statistical significance is indicated as follows: *p < 0.05, **p < 0.01, ***p < 0.001.

Nrf2 is a nuclear transcription factor crucial for cellular protection against oxidative stress. Our study observed that hypothermia at 33°C and treatment with DFO significantly increased Nrf2 protein levels in nuclear lysates while decreasing Nrf2 levels in the cytoplasm, both in vivo (Fig. 6A–D) and in vitro (Fig. 6E–H). Immunofluorescence results were consistent with Western blot findings (Fig. 6I, J). These findings indicate that hypothermia at 33°C and DFO promote Nrf2 nuclear translocation following CA and H/R, thereby facilitating Nrf2 activation.

Previous studies have highlighted NCOA4 and FPN1 as target genes regulated by Nrf2 [21]. To investigate whether Nrf2 modulates ferroptosis through NCOA4 and FPN1 during H/R injury, we employed an Nrf2 activator in H/R cell models. As is shown (Fig. 6K–P), activation of Nrf2 significantly decreased the expression of NCOA4 and 4-HNE in H/R cells while enhancing the expression of FPN1 and Fth1. Furthermore, Nrf2 activation led to a marked decrease in Fe²^+^ fluorescence intensity and MDA levels (Fig. 6Q–S). These findings suggest that Nrf2 activation may mitigate ferroptosis and by downregulating NCOA4 and upregulating FPN1 expression under conditions of H/R injury.

## Discussion

In the current study, we demonstrate that TTM at 33°C, rather than 36 °C, significantly enhances myocardial function, attenuates myocardial damage and lipid peroxidation in a rat model of CA/CPR. These effects are primarily mediated by the inhibition of ferroptosis. Our findings suggest that hypothermia at 33 °C promotes iron homeostasis by facilitating Nrf2 nuclear translocation, which subsequently regulates ferritinophagy and iron export during H/R injury. Additionally, DFO suppresses ferroptosis by activating the Nrf2 axis, in addition to its iron chelation properties.

PRMD is a reversible global dysfunction that manifests within minutes of arrest and peaks between 2 and 5 h after resuscitation. This underscores the critical importance of early intervention and treatment strategies [22]. TTM has emerged as a cornerstone in post-cardiac arrest care, with guidelines recommending maintaining a constant temperature between 32 °C and 37.5 °C during post-arres 好t temperature control [4, 23]. TTM has demonstrated significant protective effects against PRMD in clinical settings and animal models, making it a pivotal cardioprotective strategy [6, 24–26]. However, determining the optimal target temperature for different patient subgroups remains an ongoing challenge. In our study, TTM at 33°C showed superior beneficial effects on hemodynamics and myocardial dysfunction after return of spontaneous circulation (ROSC), without significant bradycardia observed. Nevertheless, TTM at 36°C did not exhibit any of these cardioprotective effects. Mild hypothermia is known to reduce metabolic rate and oxygen consumption [27] thereby protecting myocardial function and mitigating mitochondrial damage by stabilizing MMP and reducing ROS production in the CA model [5, 6, 28]. The present study demonstrated that treatment with hypothermia at 33°C showed stronger protection against myocardial damage, oxidative stress and mitochondrial injury in vivo and vitro experiments.

Ferroptosis is a newly discovered form of cell death characterized by the disruption of GXP4-dependent antioxidant defense, excessive accumulation of iron overload and lipid peroxides [9]. Our prior research has already demonstrated the involvement of ferroptosis in PRMD, and we’ve established that DFO, acting as a ferroptosis inhibitor, effectively alleviates myocardial dysfunction in this context [13]. In the current study, we delve deeper into the mechanisms underlying ferroptosis in PRMD. Central to this process is the maintenance of intracellular iron homeostasis, which hinges on the regulation of ferritin synthesis/degradation and iron export via ferroportin [29].

Ferritinophagy, facilitated by NCOA4, plays a crucial role in intracellular iron regulation by targeting FTH1, a key ferritin subunit that stores iron. This process involves NCOA4 binding to FTH1, facilitating the delivery of iron-bound ferritin to autophagosomes for degradation, thereby releasing iron into the cytoplasm [30]. Excessive ferritinophagy during I/R injury has been linked to ferrous iron overload, exacerbating lipid peroxidation [15, 31]. Inhibiting NCOA4 through knockdown or inhibition has shown promise in reducing ferritinophagy and mitigating ferroptosis by lowering intracellular labile iron pools [14, 32]. Our animal experiments revealed elevated ferrous iron and NCOA4 levels, alongside decreased GPX4 and FTH1 levels in myocardial tissue following resuscitation, which were effectively reversed by TTM at 33 °C, as validated in vivo. Subsequently, using siRNA, we demonstrated that NCOA4 knockdown reduces GPX4 expression under H/R conditions, underscoring NCOA4’s pivotal role in ferroptosis. The interaction between NCOA4 and FTH1 is essential for ferritinophagy, and our co-IP assays and immunofluorescence co-localization experiments revealed that hypothermia at 33°C disrupts this interaction, as depicted in (Fig. 5).

FPN1, the sole known mammalian iron exporter, crucially regulates iron homeostasis by facilitating iron efflux [33]. Previous studies have indicated that augmenting FPN1 expression reduces intracellular iron levels during myocardial ischemia-reperfusion injury [17]. In our current study, we observed a decline in FPN1 levels following resuscitation, potentially contributing to impaired iron transport. Treatment with TTM at 33°C, however, enhanced FPN1 expression both in vivo and in vitro. Consequently, we hypothesize that following cardiac arrest and CPR, NCOA4-mediated ferritinophagy is activated while FPN1 expression is reduced, leading to iron accumulation. Hypothermia at 33°C mitigates this process by attenuating NCOA4-mediated ferritinophagy and promoting FPN1 expression, thereby reducing cellular susceptibility to ferroptosis. These findings underscore the role of iron homeostasis regulation in the cardioprotective mechanisms of hypothermia at 33°C against ferroptosis-induced myocardial injury.

However, the precise mechanisms through which hypothermia modulates labile iron pools in cardiomyocytes during H/R injury remain elusive. Nrf2 is a well-established transcription factor known for its role in regulating genes involved in cellular redox homeostasis and ferroptosis, such as GPX4 [34]. Nrf2 activity is largely regulated by its interaction with Keap1. Under normal conditions, Nrf2 forms a complex with Kelch-like ECH-associated protein 1 (Keap1) in the cytoplasm. When oxidative stress occurs, this complex dissociates, allowing Nrf2 to translocate into the nucleus. There, Nrf2 binds to the upstream promoter region of the antioxidant response element (ARE) and small musculoaponeurotic fibrosarcoma (sMaf) protein family members, orchestrating the expression of target genes [16, 35]. In our study, we observed that hypothermia at 33°C facilitated Nrf2 nuclear translocation following cardiac arrest and H/R injury, as evidenced by Western blot and immunofluorescence analyses conducted both in vivo and in vitro (Fig. 6).

Recent studies have highlighted that genetic knockout or knockdown of Nrf2 leads to an increase in NCOA4 levels and a decrease in FPN1 expression, which heightens sensitivity to ferroptosis [17, 18, 21, 36]. To explore whether activating Nrf2 could mitigate NCOA4-mediated ferritinophagy and enhance FPN1 expression in vitro, we treated H9c2 cells with an Nrf2 activator during H/R. Remarkably, this intervention resulted in reduced NCOA4 levels and increased FPN1 expression under H/R conditions. These findings suggest that hypothermia at 33°C promotes Nrf2 activation, thereby suppressing NCOA4-driven ferritinophagy and facilitating iron export, thus regulating iron homeostasis effectively.

DFO, known for its iron-chelating properties, has been shown to exert beneficial effects in animal models of CA/ CPR and I/R injury [13, 37] In our study, DFO served as the initial positive control. Consistent with previous research, we found that DFO improved PRMD, attenuated myocardial damage and mitochondrial injury, and notably decreased ferrous iron levels in cardiomyocytes. Accumulating evidence suggests that DFO also suppresses ferroptosis by activating the Nrf2 pathway, promoting the translocation of Nrf2 into the nucleus across various diseases [38, 39]. However, there has been limited exploration of this relationship in CA/CPR scenarios. In our current investigation, we were surprised to discover that treatment with DFO not only enhanced Nrf2 activation but also regulated downstream effectors including NCOA4, FTH1, and FPN1, both in animal models and cell cultures. These findings underscore how DFO suppresses ferroptosis through multiple mechanisms: by chelating iron and activating the Nrf2 axis. This dual action highlights DFO as a promising therapeutic candidate for mitigating ferroptosis-mediated damage in PRMD.

In conclusion, our study demonstrates that TTM at 33°C, rather than 36°C, significantly enhances post-resuscitation myocardial function and reduces myocardial damage by mitigating ferroptosis. These protective effects are primarily mediated through the activation of Nrf2 and the regulation of iron homeostasis. Furthermore, DFO complements these effects by not only chelating iron but also activating the Nrf2 pathway to suppress ferroptosis.

## Methods

### Animal preparation

All procedures involving animals were approved by the Committee on Ethics of animal Experiments at the Anhui Medical University (LLSC 20210347) and performed in compliance with the relevant ethical guidelines. Thirty healthy male Sprague-Dawley male rats weighing 450–550 g were provided by the laboratory animal center of Anhui Medical University. The sample size was established based on previous studies, the complexities involved in model construction, and preliminary experimental data. Before the procedure, animals were fasted for 12 h overnight, but allowed free access to water. Anesthesia was induced by inhalation of carbon dioxide (CO_2)_ for 20–30 s to induce anesthesia, followed by intraperitoneal pentobarbital (45 mg/kg). Additional doses (10 mg/kg) were administered per hour or as required to maintain anesthesia. The trachea was orally intubated with a 14 G cannula mounted on a blunt needle (Abbocath-T; Abbott Hospital Products Division). Mechanical ventilation was initiated using a multichannel ventilator (KW-100-2, NJKEWBIO, China) with a tidal volume of 0.6 mL/100 g body weight at a F_IO2_ of 0.21, and end-expiratory CO_2_ (ET CO_2_) was continuously recorded. Lead II electrocardiogram (ECG) was recorded continuously monitored. Two PE-50 catheters (PE50, Smith Medical, United Kingdom) was placed into the left femoral artery, the left veins, respectively. The artery catheter was used to monitor arterial pressure, the left femoral catheter for fluids and drugs injection. A thermocouple microprobe (IT-18; Physitemp Instruments, Clifon, NJ) was placed into the right femoral vein to detect temperature. The core temperature was maintained at a certain level for 4 h after ROSC by a heat lamp and an animal water bath insulation system (category NO.2020, YUYAN INSTRUMENTS, CHINA).

### Animal model of cardiac arrest

**Fig. 7:**
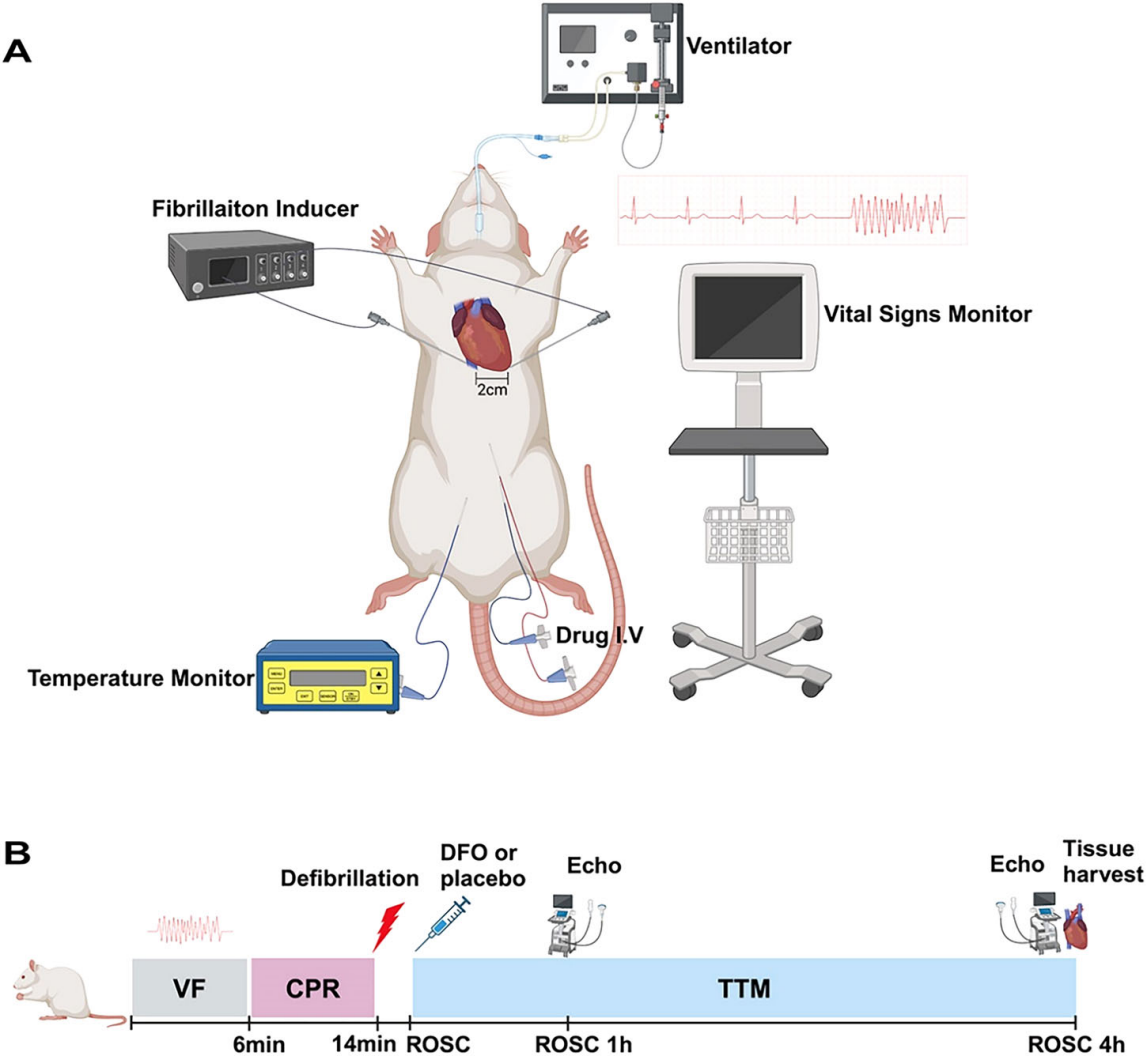
Experimental procedure. **A** Experimental devices. The rat is linked to a ventilator to maintain respiration, and a fibrillation inducer is used to induce cardiac fibrillation, as shown by the fibrillation waveform on the ECG. A temperature monitor tracks the rat’s body temperature, and a drug I.V. is used for administering medication. A vital signs monitor records the rat’s vital signs data. The heart area of the rat is marked with a 2 cm scale to indicate the precise location for experimental procedures. **B** VF ventricular fibrillation, CPR cardiopulmonary resuscitation, TTM targeted temperature management, ROSC Return of Spontaneous Circulation, DFO deferoxamine.

Baseline vital (temperature, heart rate and arterial pressure) and echocardiography were measured 15 mins before induction of ventricular fibrillation (VF). The rat model of CA was produced as described previously [40]. Briefly, VF was induced by electrical stimulation from two acupuncture needles inserted into epicardium. One needle was inserted into the apex of heart at the strongest pulsation point (point A) at an angle of 60°. The other needle was inserted into point B, ~2.0 cm horizontally to the right of point A, at an angle of 75°. The insertion depth is ~1.5–2.0 cm. When the needle tip reached the epicardium, the needle tail exhibited swinging synchronized with the rat’s heart rate, resulting in transient ventricular arrhythmias observed on the ECG. Transient drop in blood pressure may also occur. VF was induced by two acupuncture needles stimulation with an initial 0.5 mA alternating current, increasing if necessary to a maximum of 1.5 mA. The stimulation was continued for 3 min to prevent spontaneous defibrillation. Mechanical ventilation was suspended once VF onset. After 6 min of untreated VF, mechanical ventilation (with a tidal volume of 0.6 mL/100 g body weight at a F_IO2_ of 1.0) was restarted, and precordial chest compression (compression rate of 200 times/min, compression depth of 1.0–1.3 cm) was initiated using a small animal cardiopulmonary resuscitation instrument (category NO. KW-XF, NJKEWBIO, China), maintained for 8 min. (Fig. 7A)

Defibrillation was immediately performed following the cessation of compressions with up to 4 J countershocks. If resuscitation was not successful after the electrical shock, precordial compression was resumed for 30 s, followed by another sequence of defibrillation, with no more than 3 cycles allowed. Return of spontaneous circulation (ROSC) was defined as the return of supraventricular rhythm with a mean arteria pressure over 50 mmHg for 5 min. The F_iO2_ of 1.0 was maintained for 1 h, then adjusted to 0.5 for the second hour, and subsequently reduced to 0.21.

### Animal randomization and intervention

Thirty male rats were randomly divided into five groups (*n* = 6): (1) sham; (2) normothermia control (NTC); (3) Deferoxamine -normothermia (DFO); (4) TTM-33 °C (33 °C); (5) TTM-36 °C (36 °C). In the sham group, animals underwent the same procedures as the other groups, except for VF and CPR, while maintaining a core temperature of 37.5 °C ± 0.2 °C for 4 h. In the NTC group, the and the core temperature was maintained at 37.5 °C ± 0.2° C for 4 h following ROSC. In the DFO group, animals were administered with DFO (200 mg/kg body weight, category NO. HY-B0988, MedChemExpress, USA) immediately upon ROSC, while maintaining a core temperature of 37.5 °C ± 0.2 °C for 4 h following ROSC. In the 33 °C group and 36 °C group, a core temperature was maintained at 33° C ± 0.2 °C and 36 °C ± 0.2 °C, respectively, for 4 h following ROSC. Saline placebo was intravenous injected (200 mg/kg body weight) immediately upon ROSC in the sham, NTC, 33 °C and 36 °C groups. Animals were sacrificed and heart tissues were obtained at 4 h after ROSC. The hearts tissues were appropriately disposed of for further assays (Fig. 7B).

### Measurements

#### Post-resuscitation hemodynamics

ECG, heart rate (HR), systolic blood pressure (SBP), mean arteria pressure (MAP), and core temperature were continuously monitored and recorded hourly following ROSC using a PC-based biological signal acquisition and processing system (Medlab-U/4C501H, NJKEWBIO, China). Doppler echocardiographic examination (6LAB, VINNO, China) was performed to calculate left ventricular ejection fraction (LVEF) at baseline, 1 h and 4 h after ROSC.

### **Histology of myocardium**

The fresh myocardial tissues were fixed with 10% paraformaldehyde, embedded in paraffin, and cut into 5 µm of thin-sections, which were placed on slides for hematoxylin-eosin staining (H&E) for assessing the morphological changes and damage degree. Then, the sections on slide glass were observed with an optical microscopy (BH-Z; Olympus Corporation, Japan).

### Transmission electron microscopy (TEM)

Samples of myocardium or cells were swiftly extracted from the left ventricle (1 mm × 1 mm × 1 mm) or collected and immediately fixed in 2.5% phosphate-glutaraldehyde. Subsequently, the samples underwent dehydration through a graded ethanol series, followed by embedding, slicing into 50–80 nm thick sections using an ultramicrotome, and staining with 5% uranyl acetate and lead citrate. Finally, the sections were observed and imaged using a Hitachi transmission electron microscope (TEM, JEM-1400, Jeol, Tokyo, Japan). The ultrastructural damage of mitochondria was evaluated based on the Flameng score.

### Malondialdehyde (MDA) and ferrous iron assays of myocardium

The fresh tissues of myocardium were perfused with PBS to remove blood and clots. After tissues weighing, they were homogenized in slurry medium. The MDA content was quantified using MDA Colorimetric Assay Kit (Elabscience E-BC-K025-M, Wuhan, China), while the determination of Fe ^2+^ level was conducted using Ferrous Iron Colorimetric Assay Kit (category NO. E-BC-K773-M, Elabscience Wuhan, China), following to the manufacturer’s instructions.

### Elisa

The levels of creatine kinase-MB (CK-MB), TNNI3/Cardiac Troponin I (cTnI) and brain natriuretic peptide (BNP) in heart tissues were measured with ELISA kit in following the manufacturer’s instructions (category NO. E-EL-R1327, Elabscience; category NO.E-EL-R1253, Elabscience; category NO.E-EL-R0126), respectively. Tissue pieces were weighed and then homogenized in PBS (tissue weight (g): PBS (mL) volume = 1:9). Enzymatic activity was assessed spectrophotometrically in absorbance at 450 nm using a 96-well plate reader.

### Cell culture and in vitro treatment

H9c2 cells, a cardiac cell strain derived from the S-D Rat left ventricle, were obtained from Oricells Biotechnology (Shanghai) Co. after STR analysis and mycoplasma detection. The cells were cultured in Dulbecco’s Modified Eagle’s Medium/Nutrient Mixture F-12 (DMEM/F12, Thermo Fisher Biochemical Products, Beijing, China), supplemented with 10% fetal bovine serum (FBS; Hyclone, Logan, UT, USA), 100 U/ml penicillin, and 100 mg/ml streptomycin in a normoxic incubator (5% CO_2_ and 95% air) at 37 °C. To induce hypoxia/reoxygenation (H/R) injury, cells were subjected to a hypoxic chamber at 37 °C with an atmosphere of 1% oxygen, 94% nitrogen, and 5% CO_2_ for 12 h. To simulate different temperature of therapeutic hypothermia (TH), the cells after hypoxia were subsequently incubated in a normoxic incubator at 37 °C, 33 °C or 36 °C for 4 h each. DFO (category NO. HY-B0988, MedChemExpress, USA) was applied to the cells at the concertation of 60 u/M at the onset of reoxygenation for 4 h in a normoxic incubater (5% CO_2_ and 95% air) at 37 °C.

### Cell viability assay

Cell viability was evaluated using a CCK kit-8 (category NO. GK10001, GloBio, USA) according to the manufacturer’s instructions. H9C2 cells were seeded in 96-well plates and allowed to reach 80% confluence. Subsequently, each well received 10 µL of CCK-8 reagent mixed with 100 µL of culture medium, followed by incubation for 2 h at 37 °C. The optical density (OD) of each well was measured at a wavelength of 450 nm using a microplate reader.

### Measurement of Reactive oxygen species (ROS) and Mitochondrial membrane potential (MMP)

Intracellular ROS levels were assessed using DCFH-DA fluorescent probe (category NO. E004-1-1, Nanjing Jiancheng Bioengineering Institute, China), following the manufacturer’s instructions. H9C2 cells were seeded in 6-well plates at a density of 5 × 10^4^ cells per well. The cells were then washed three times with PBS and incubated with 10 μM DCFH-DA for 30 min at 37 °C in a light-free environment. Following the incubation, the cells were examined under an inverted fluorescence microscope (Axio Vert.A1, Olympus), and the fluorescence images indicating intracellular ROS levels were quantified using the Image J software. The MMP was evaluated using JC-1 fluorescent probe (category NO. C2006, Beyotime, China). Following the manufacturer’s instructions, JC-1 staining working solution was carefully mixed with the cells. Subsequently, the cells were then incubated at 37 °C for 30 min in a cell culture incubator. After two washes with JC-1 buffer solution, the cells were supplemented with culture medium and examined and imaged under an inverted fluorescence microscope (Axio Vert.A1, Olympus), and JC-1 red/green fluorescence ratios were quantified using the Image J software.

### Assessment of cell ferrous iron (Fe 2 + ) fluorometric assay

FerroOrange (category NO. E-BC-F 101, Elabscience, China) is a fluorescent probe utilized for measuring intracellular Fe ^2+^ levels. Cells were incubated with working solution at 37 °C for 30 min, and images were captured using an inverted fluorescence microscope (Axio Vert.A1, Olympus) at excitation of 542 nm and emission of 575 nm.

### **Immunofluorescence and immunofluorescence colocalization**

The cells were fixed with 4% paraformaldehyde for 15 min and bathed with PBS. Subsequently, the cells were permeabilized with 0.5% Triton X-100 at room temperature for 10 min. Blocking was performed using, followed by overnight incubation with primary antibodies: anti-FTH1(1:500; GTX101733, Gene Tex), anti-NCOA4 (1:500, DF4255, Affinity), anti-Nrf2 (1:100, 6396-1-AP, Proteintech), anti-FPN1(1:100, 26601-1-AP, Proteintech) at 4°C. After washing with PBS, excess liquid was removed using absorbent paper, and the cells were then incubated with Cy3-labeled Goat Anti-Rabbit IgG secondary antibody (Boster, China) at 37 °C for 1 h. Nuclei were counterstained with DAPI (Beyotime Institute of Biotechnology, China) for 5 min. Images were acquired using with Zeiss Confocal Microscope. Pearson’s coefficient values for signal overlap were determined using ImageJ [National Institutes of Health (NIH)].

### SiRNA-mediated knockdown of NCOA4

To knock down protein levels of NCOA4, 1 × 10^5^ cells were seeded in six-well plates and, 24 h later, treated with 5 nM nontargeted control (control), NCOA4 siRNA using Lipofectamine 2000 according to the manufacturer’s instructions. Three different siRNA constructs against each target of interest were obtained from Shanghai GenePharma Co. Ltd, Shanghai, China (Table S1). The construct that achieved the highest level of knockdown was selected for further study (Fig. S1).

### Western blot analysis

Nuclear proteins and cytoplasmic proteins were extracted from fresh heart tissues or cells using nuclear protein and cytoplasmic protein extraction kit (category NO. P0028, Beyotime, China). Total proteins were extracted from tissues or cells by the mixture of RIPA lysate (category NO. P0013B, Beyotime, China) and phosphatase and protease inhibitor cocktails (Roche, 5892970001), and separated on a 10% Tris·HCl Ready Gel (Bio-Rad) and transferred to a PVDF membrane, before blocking with 5% skimmed milk powder in TBS-T (TBS plus 0.1% Tween 20). Membranes were then incubated with antibodies against GPX4 (1:1000, rabbit, ab125066,Abcam), 4-HNE(1:1000, rabbit, ab46545, Abcam), NCOA4 (1:1000, rabbit, ab314553, Abcam), FTH1 (1:1000, rabbit, GeneTex, GTX101733), Nrf2 (1:1000, rabbit, 60004–1-Ig Proteintech), β-actin (1:1000, rabbit, 81115-1-RR, Proteintech), Histone H3(1:1000, 17168-1-AP, Proteintech) overnight at 4 °C. Next, membranes were incubated with goat anti-rabbit IgG (H + L) (1:10000, CST, #5151 P, USA) or anti-mouse IgG (H + L) (1:10000, CST, #5257 P, USA) secondary antibody diluted with secondary antibody diluent (1:1000, P0023D, Beyotime,). Protein bands were detected with an Odyssey color infrared laser scan-imaging instrument (Li-Cor, USA) and analyzed using Image J software. Data were recorded as relative density ratios.

### Co‐immunoprecipitation (IP) assay

The concentration of protein in H9c2 cells was assessed by the BCA protein assay kit (category NO. P0012, Beyotime, China). The cells were lysed in mixed buffer (50 mmol/L Tris–HCl, 150 mmol/L NaCl, 1% NP-40, 1 mmol/L NaF, 1 mmol/L EDTA, 1 mmol/L PMSF, 1 mmol/L Na3VO4, and protease inhibitor cocktail). The protein supernatant was hatched with 1–2 mg rabbit polyclonal IgG control antibody and 25 ml resuspended volume of protein A/G plus agarose (Beyotime, P2055) for 1 h. Then, the protein supernatant was incubated with 1 ml anti-NCOA4 (Affinity Biosciences, DF4255, 1:1,000) for 24 h at 4°C. The cells were cultured again lasting 2 h. The immunoprecipitation buffer was washed several times, and 50 ml of SDS-PAGE loading buffer was added and then denatured. Finally, the co-IP assay was performed.

### Statistical analysis

There is no ramdomization or blinding in this study. Data were analyzed using SPSS 26.0 and expressed as means ± standard error of the mean (S.E.M). The Shapiro-Wilk test was applied to evaluate the normality of the data distribution. Group comparisons were conducted using one-way analysis of variance (ANOVA) with post-hoc analysis by Tukey’s test for pairwise comparisons and Bonferroni’s correction for multiple comparisons. *p*-value < 0.05 was defined as statistically significant.

## Supplementary information

Supplementary material

Original Data

## Data Availability

The datasets generated and analyzed during the current study are available from the corresponding author on reasonable request.
