# Supplementary material for: Targeted temperature management alleviates post-resuscitation myocardial dysfunction by inhibiting ferroptosis
Source: Cell Death Discov. 2025 Feb 21;11:71. doi: 10.1038/s41420-025-02356-5 (PMC11845627; doi:10.1038/s41420-025-02356-5)
Supplement: Supplementary file 1 — Supplementary material [file 41420_2025_2356_MOESM1_ESM.docx]

| **SiRNA** | **Sequence** |  |
| --- | --- | --- |
| **NCOA4-1** | Sense | GCCCUACAAUGUGAAUGAUTT |
|  | Anti-sense | AUCAUUCACAUUGUAGGGCTT |
| **NCOA4-2** | Sense | GGAGAACAGUCAGACUUCUTT |
|  | Anti-sense | AGAAGUCUGACUGUUCUCCTT |
| **NCOA4-3** | Sense | CCUUGUCAGAGUGGCUUAUTT |
|  | Anti-sense | AUAAGCCACUCUGACAAGGTT |
| **Negative control FAM** | Sense | 5'UUC UCC GAA CGU GUC ACG UTT-3 |
|  | Anti-sense | 5'-ACG UGA CAC GUU CGG AGA ATT-3' |

**Table S1. siRNA sequence of NCOA4.**

This table presents the siRNA sequences designed to silence NCOA4. It includes three specific siRNAs (NCOA4-1, NCOA4-2, NCOA4-3) along with a negative control siRNA labeled with FAM. For each siRNA, both the sense and antisense strands are provided. The negative control siRNA serves as a reference for baseline comparisons in the experiments.


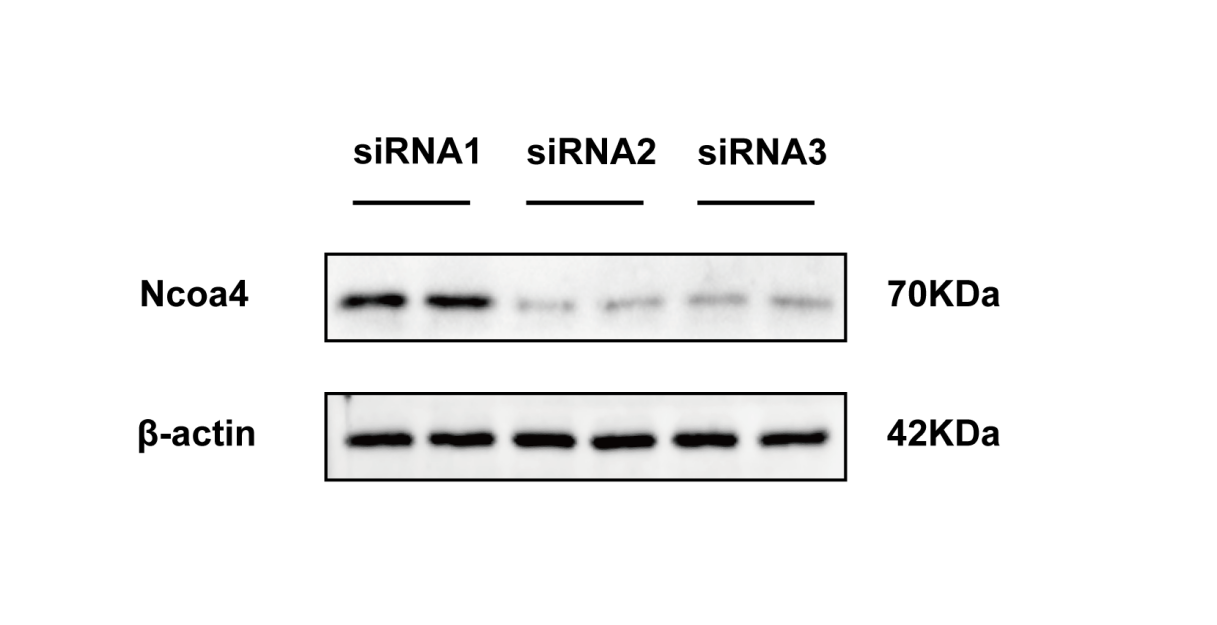


**Fig.S1 Validation of NCOA4 knockdown efficiency by siRNA**

Western blot analysis demonstrating the knockdown efficiency of NCOA4 by three specific siRNAs (siRNA1, siRNA2, and siRNA3).
