# Supplementary figures and images for: Targeted temperature management alleviates post-resuscitation myocardial dysfunction by inhibiting ferroptosis
Source: Cell Death Discov. 2025 Feb 21;11:71. doi: 10.1038/s41420-025-02356-5 (PMC11845627; doi:10.1038/s41420-025-02356-5)

Figuer 3H

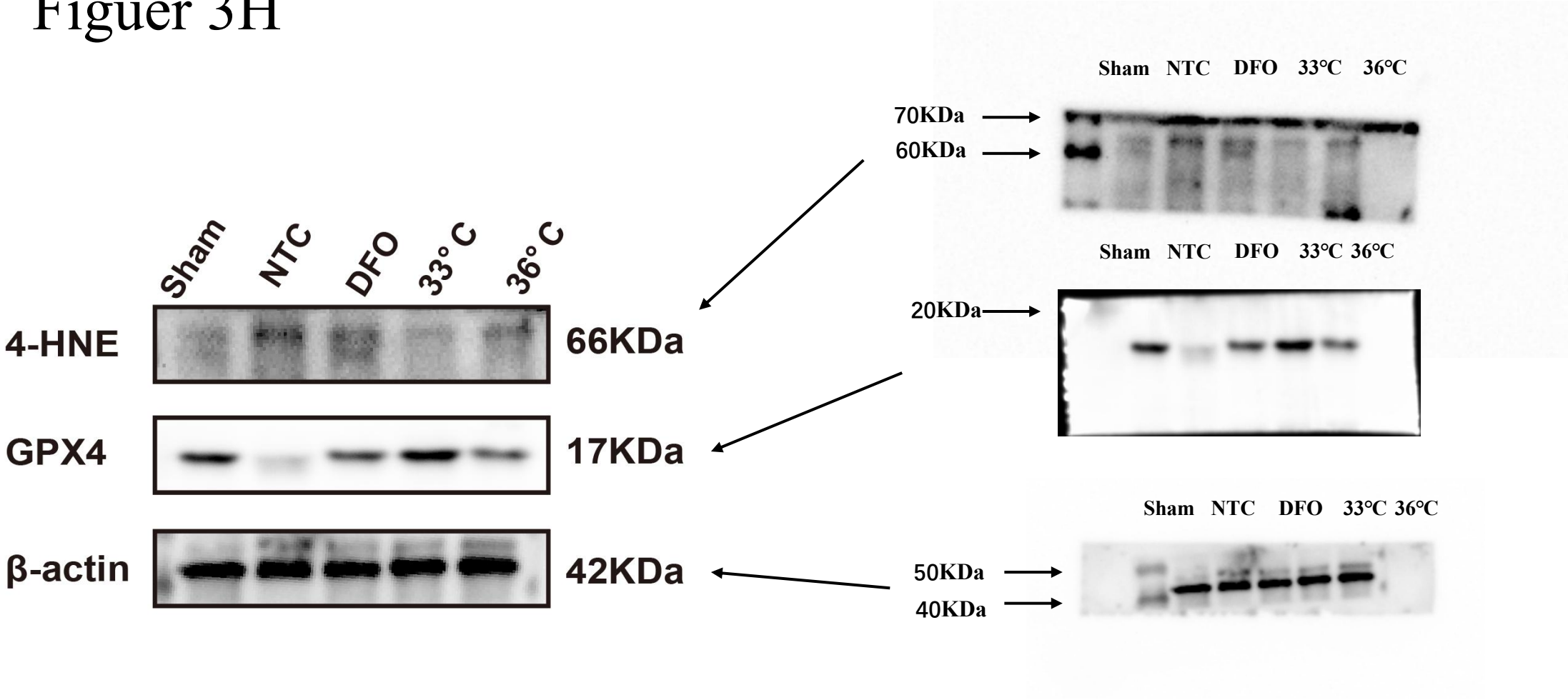

Figuer 3K

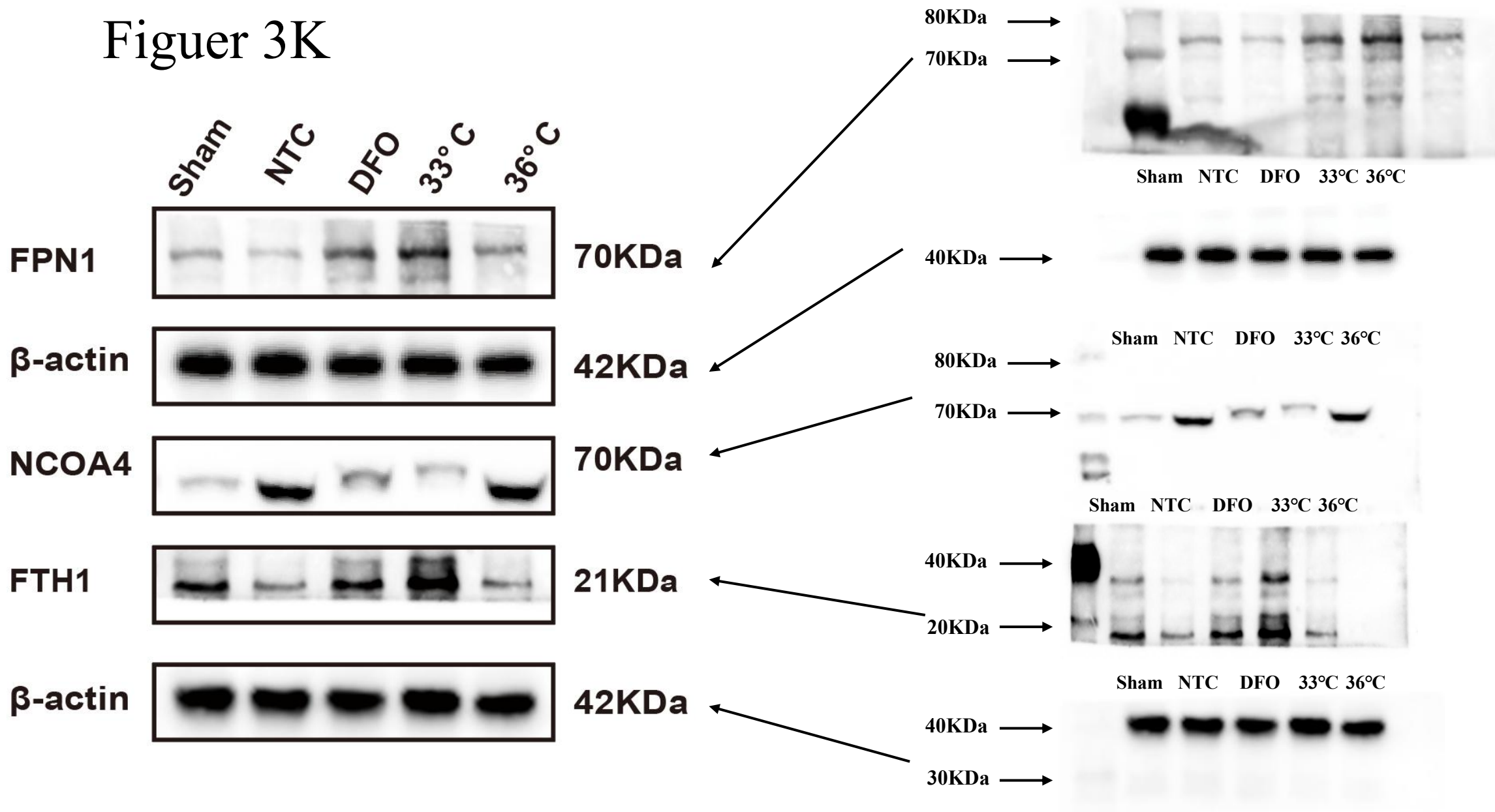

Figuer 5C

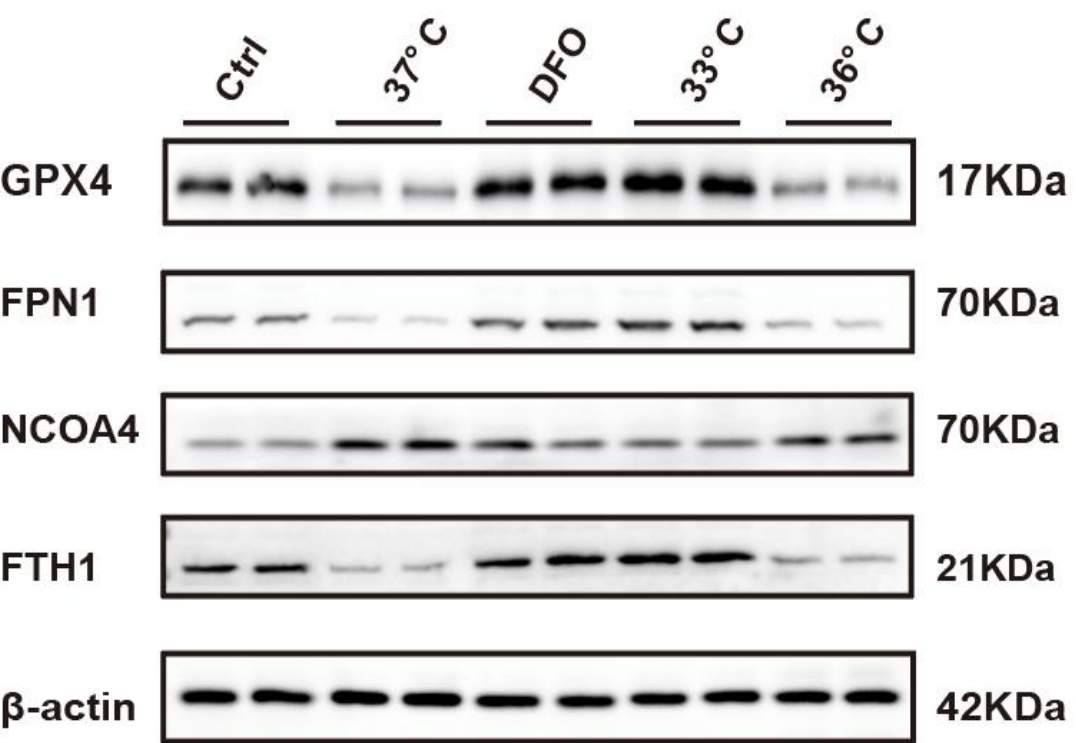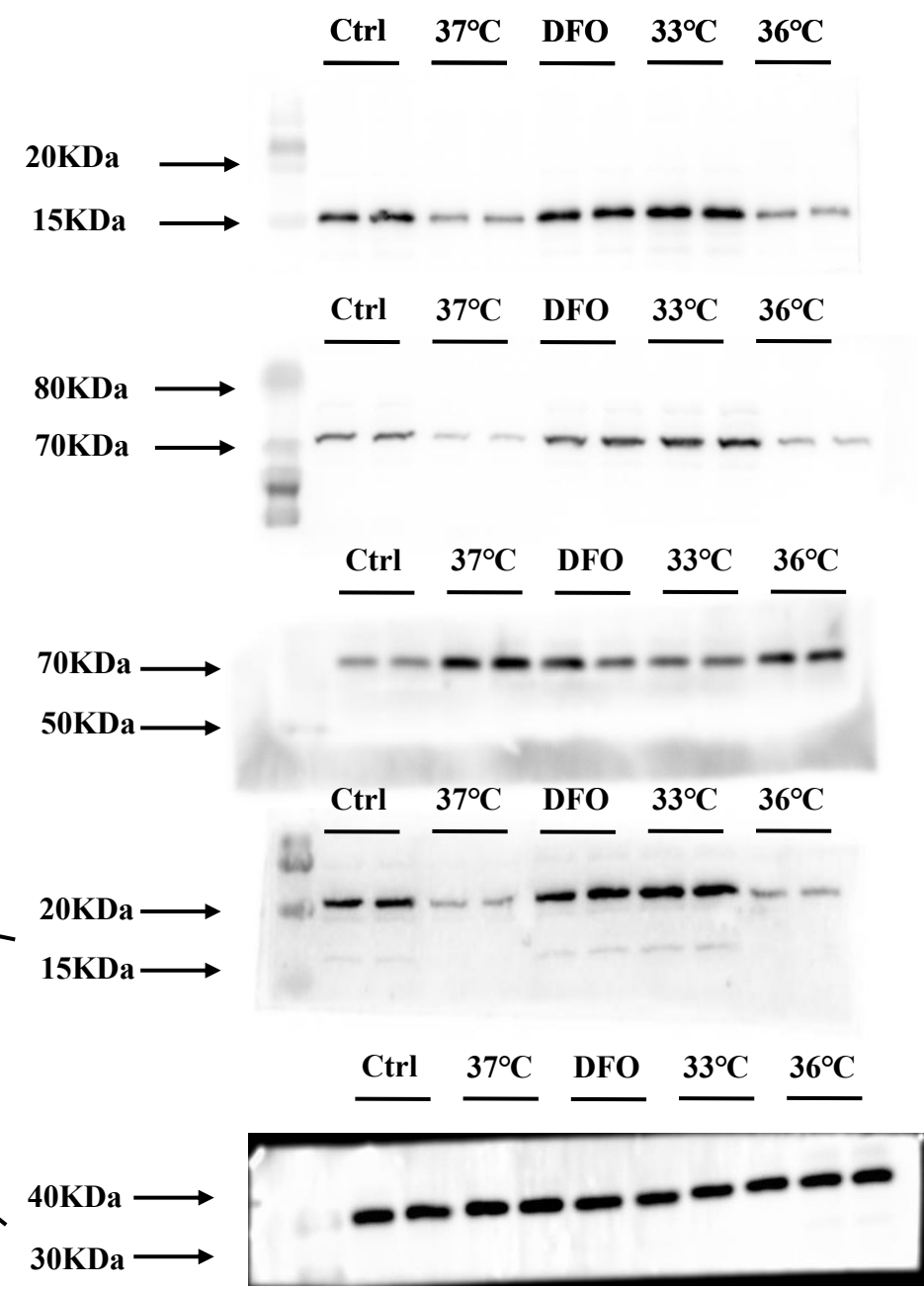

Figuer 6E

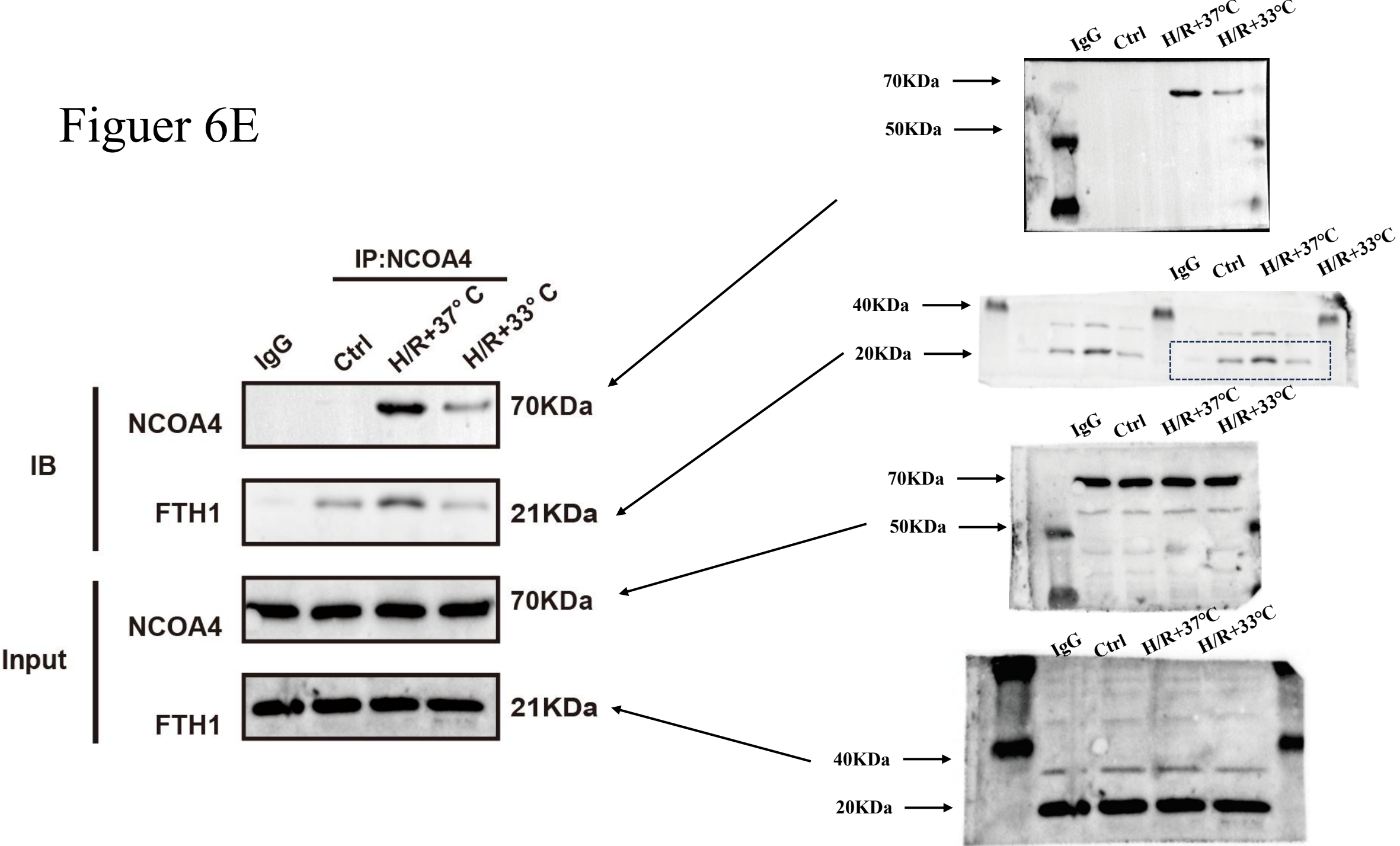

Figuer 6H

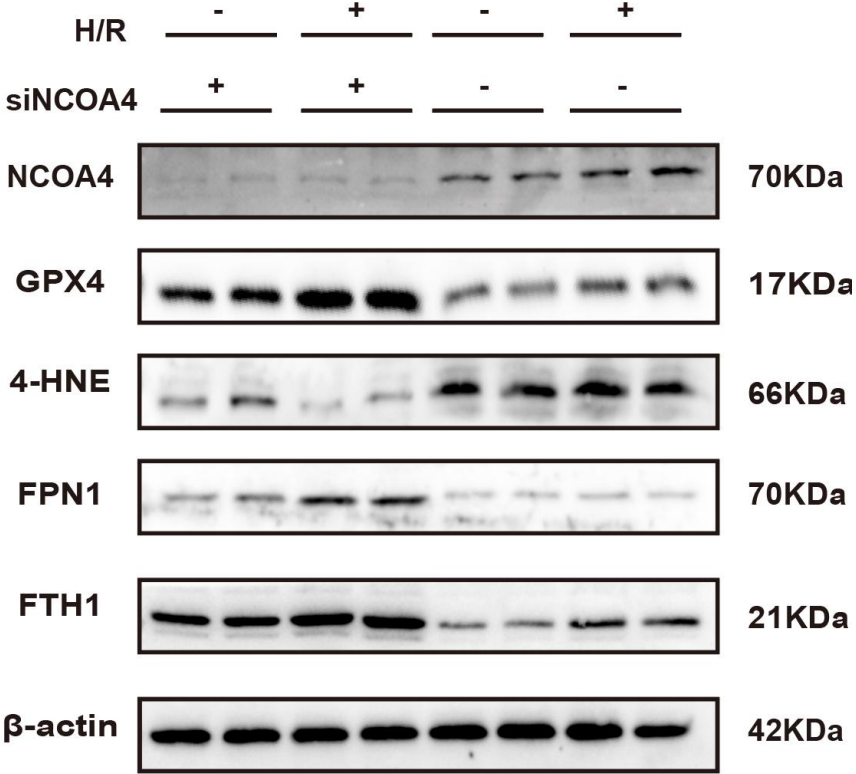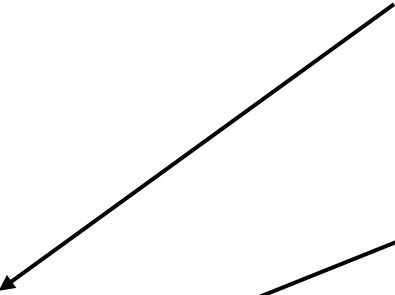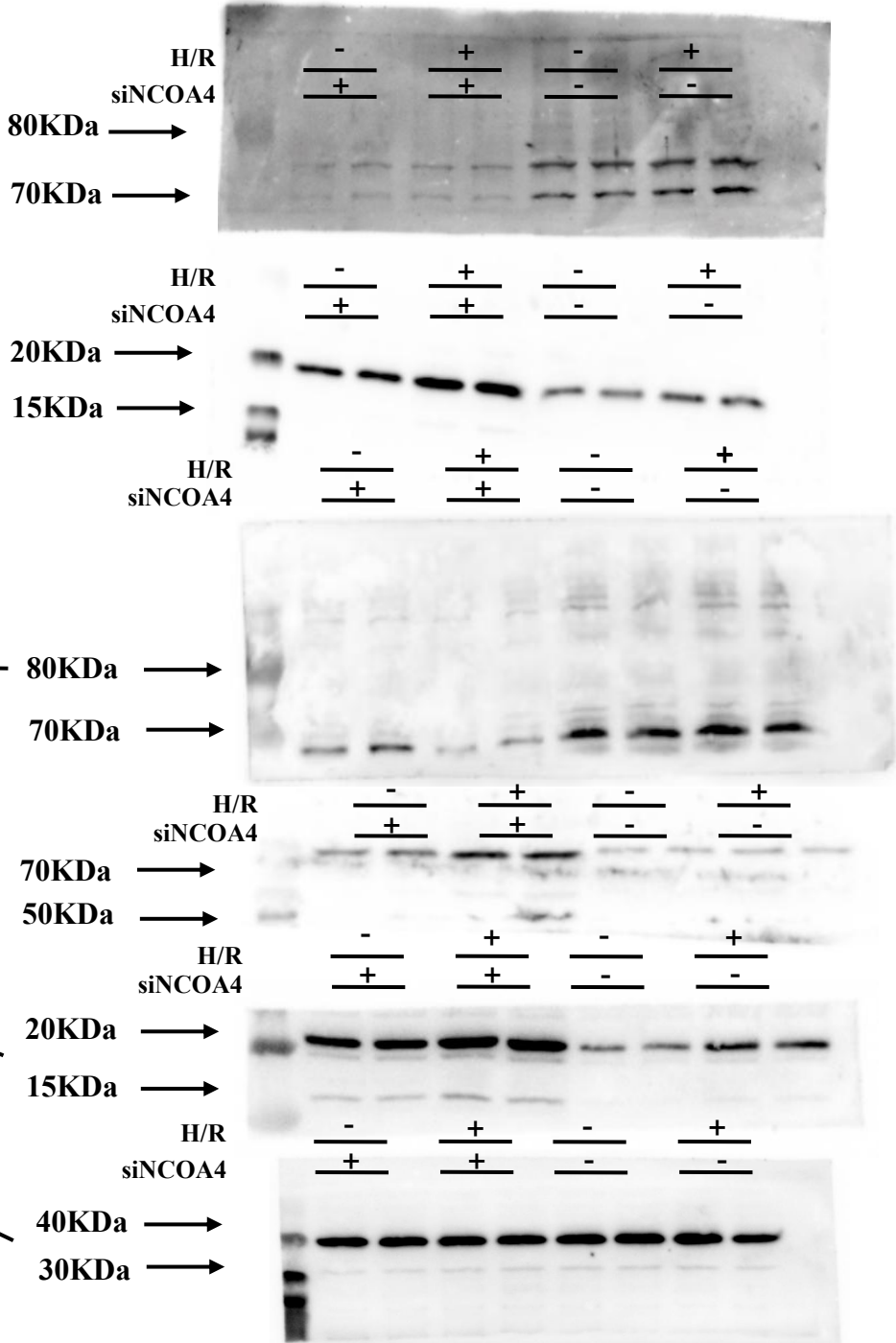

Figuer 7A

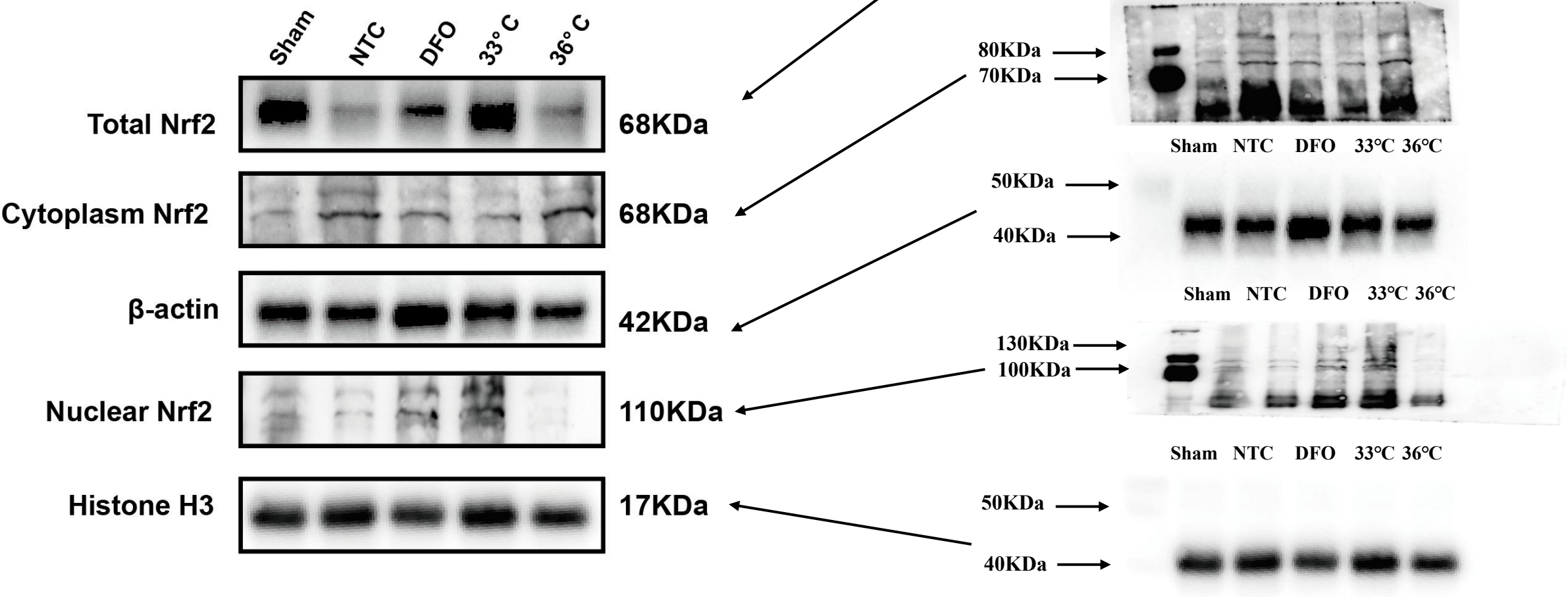

Figuer 7E

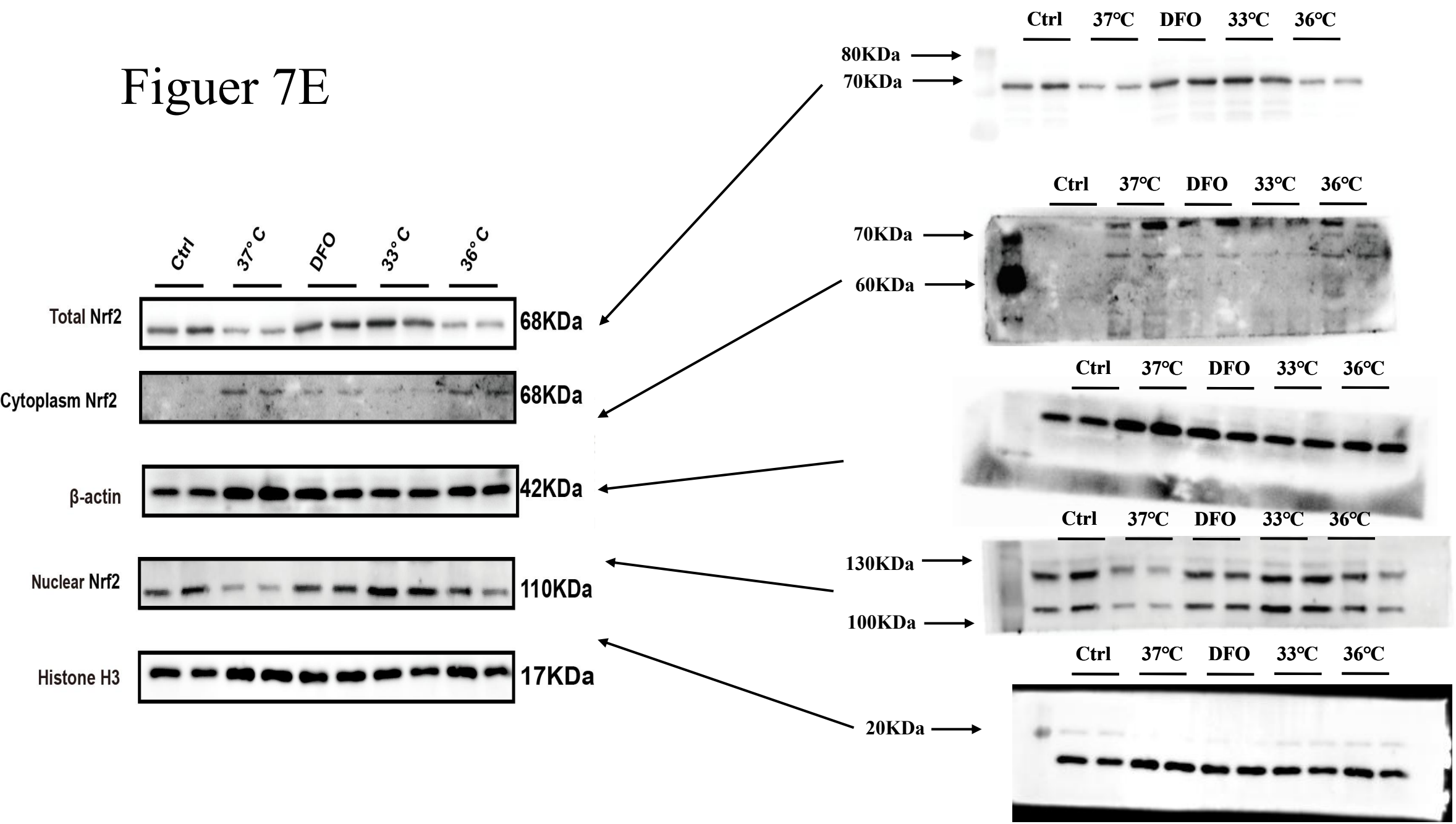

Figuer 7K

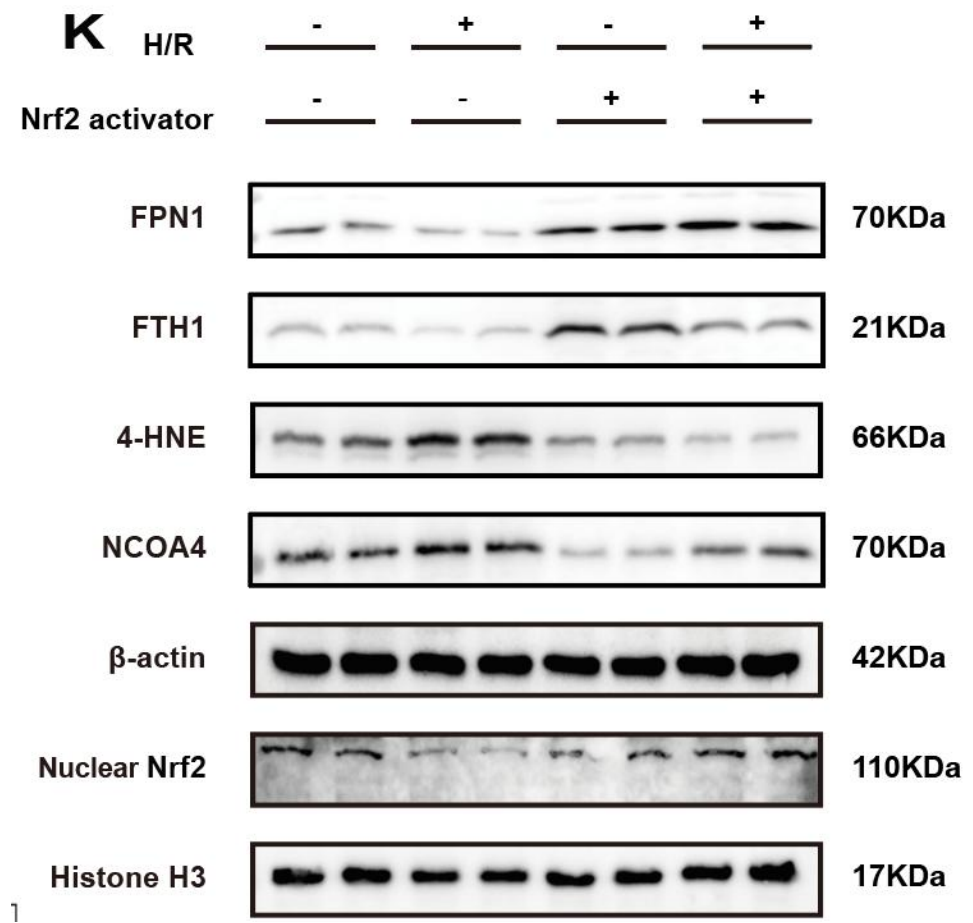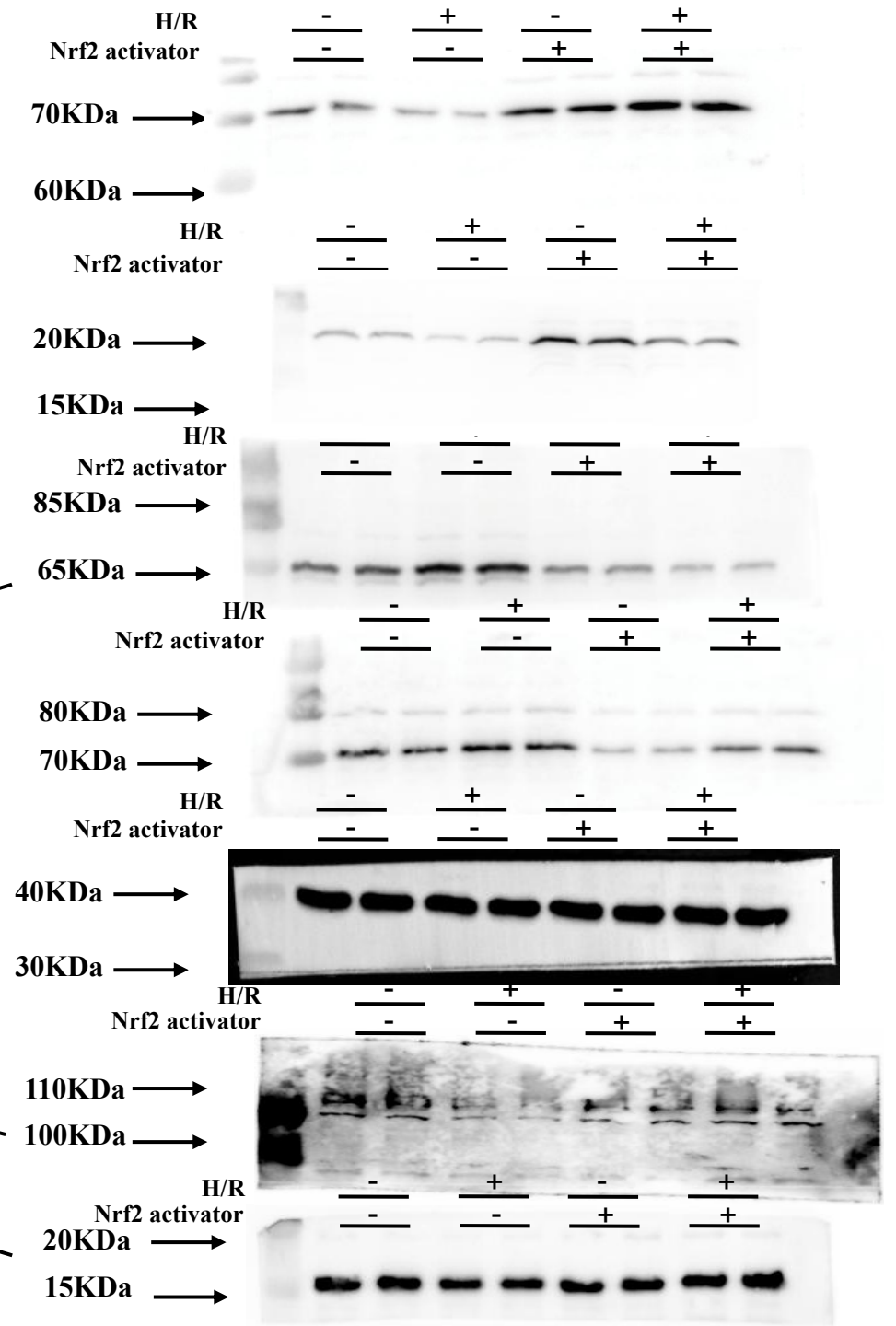

# Figuer supplement 1

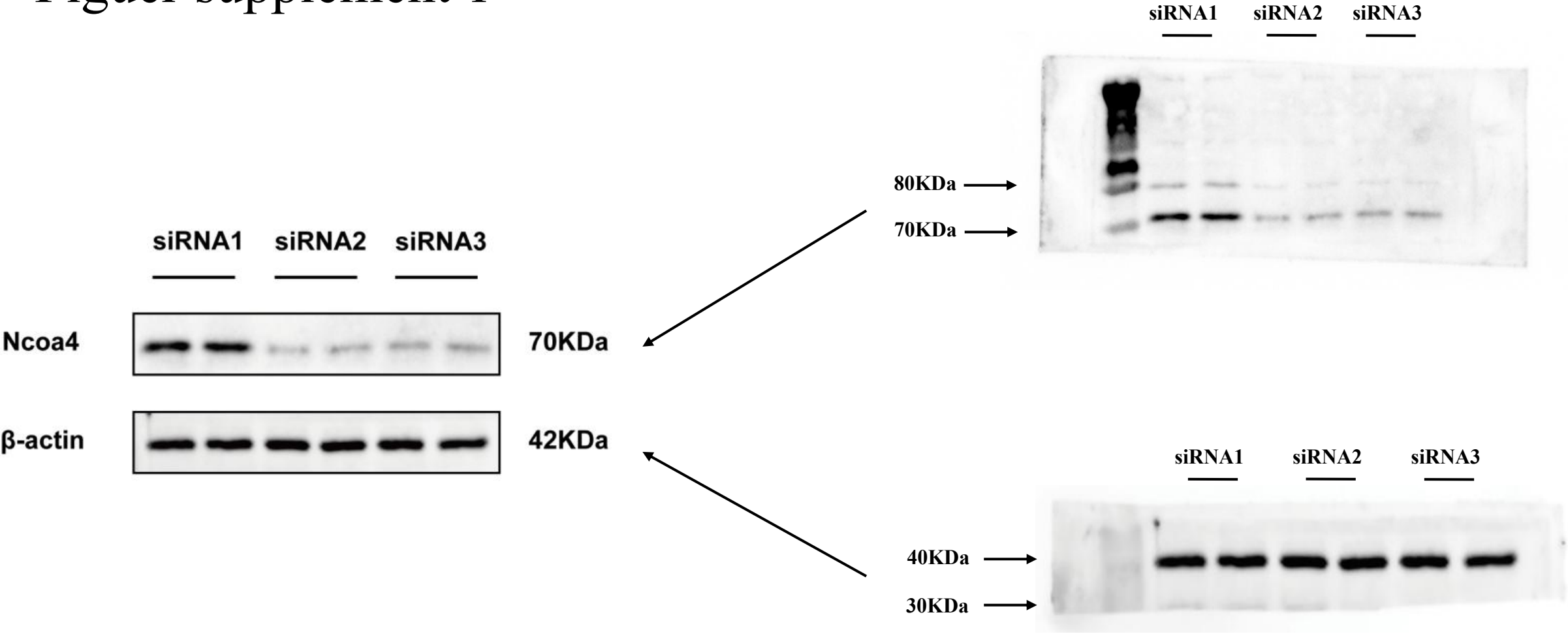

Supplement: Supplementary file 2 — Original Data [file 41420_2025_2356_MOESM2_ESM.pdf]
